# Catechol-*O*-methyltransferase Inhibitors from *Calendula officinalis* Leaf

**DOI:** 10.3390/molecules28031333

**Published:** 2023-01-30

**Authors:** Wataru Kadowaki, Ryo Miyata, Misa Fujinami, Yoshizumi Sato, Shigenori Kumazawa

**Affiliations:** 1 Graduate School of Integrated Pharmaceutical and Nutritional Sciences, University of Shizuoka, 52-1 Yada, Suruga-ku, Shizuoka 422-8526, Japan; 2 Kisho Corporation Co., Ltd., 3-4-27 Kitasuna, Koto-ku, Tokyo 136-0073, Japan

**Keywords:** *Calendula officinalis*, flavonoid, phenylpropanoid, catechol-*O*-methyltransferase, Parkinson’s disease

## Abstract

*Calendula officinalis* is commonly known as marigold and its flowers are used in herbal medicines, cosmetics, perfumes, dyes, pharmaceutical preparations, and food products. However, the utility of its leaves has not been studied in depth. The purpose of the present study was to identify the major compounds in *C. officinalis* leaves and to determine the inhibitory properties of the isolated compounds toward human catechol-*O*-methyltransferase (COMT), a key neurotransmitter involved in Parkinson’s disease and depression. We isolated and identified ten compounds, including two phenylpropanoids and seven flavonoids, from *C. officinalis* leaf extracts, of which four flavonoids were identified from *C. officinalis* leaves for the first time. Eight compounds exhibited COMT inhibitory activities with IC_50_ values of less than 100 μM. Our results indicate that compounds in *C. officinalis* leaves are potentially effective for preventing Parkinson’s disease and depression. Thus, *C. officinalis* leaves may hold promise as dietary supplements.

## 1. Introduction

*Calendula officinalis* is a medicinal plant belonging to the Asteraceae family and is distributed mainly over large areas of the Mediterranean [1,2]. The petals of *C. officinalis* exhibit a broad range of biological activities, including antioxidant [3], antitumor [4,5], antibacterial [4], and anti-inflammatory activities [5,6]. Oleanane-type triterpene glycosides [5,7], triterpene alcohols [8,9], flavonoid glycosides [5], and carotenoids [10] have been found in the petals of *C. officinalis* and contribute to various biological activities. The petals of *C. officinalis* have been used in Europe since the 13th century for treating wounds, and a variety of cosmetics have been developed from the plant [11,12].

Although *C. officinalis* petals are used medicinally, the other parts of the plant are presently not utilized. Several recent studies have drawn attention to the effective use of waste- or by-products arising from productization processes [13,14]. Effective utilization of the currently unused parts of *C. officinalis* requires information on their chemical composition and biological activities. Although the leaves of *C. officinalis* are used as a traditional treatment for varicose veins in India [1], other biological uses have not been investigated. We therefore investigated the usability of *C. officinalis* leaves by isolating the major components and determining their structures by spectroscopic analysis. In addition, the catechol-*O*-methyltransferase (COMT) inhibitory activities of both the leaves of *C. officinalis* and the isolated compounds were evaluated. COMT is a target enzyme of Parkinson’s disease and depression [15,16]. Current anti-parkinsonian drugs exhibit severe toxicity, whereas natural sources could provide many potentially safe COMT inhibitors [17]. The leaves of *C. officinalis* and the isolated compounds exhibited remarkable COMT inhibitory activities, indicating their utility as bioactive ingredients toward Parkinson’s disease.

## 2. Results and Discussion

### 2.1. Total Polyphenol Content and COMT Inhibitory Activity of C. officinalis Leaves

**Figure 1:**
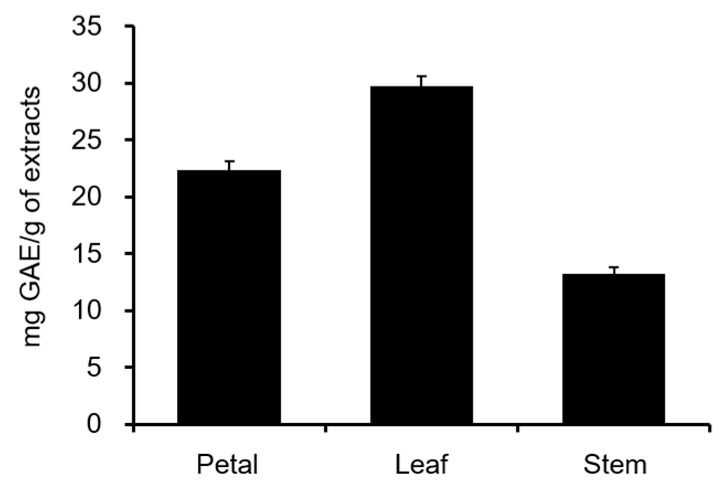
Total polyphenol content of ethanol extracts from each part of *C. officinalis* as determined by the Folin–Ciocalteu colorimetric method. GAE means gallic acid equivalent.

**Figure 2:**
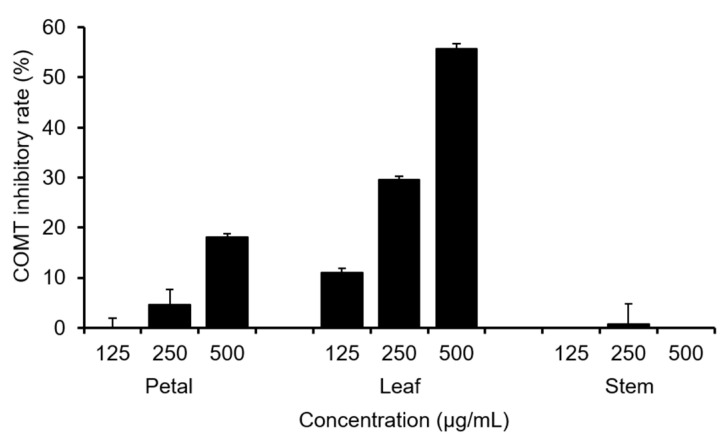
Catechol-*O*-methyltransferase (COMT) inhibitory activity of ethanol extracts of *C. officinalis* petals, leaves, and stems.

First, we compared the total polyphenol content of each part of *C. officinalis* (petal, leaf, and stem) using the Folin–Ciocalteu colorimetric method. As shown in Figure 1, the leaves showed the highest content. Next, the COMT inhibitory activities of various parts of *C. officinalis* were evaluated (Figure 2). Consistent with the total polyphenol content, the ethanol extracts of leaves exhibited the highest activity and were three times that of the petals, suggesting that *Calendula* leaves can be used as a source of natural compounds effective toward Parkinson’s disease.

### 2.2. Component Analysis of the Leaves of C. officinalis

**Figure 3:**
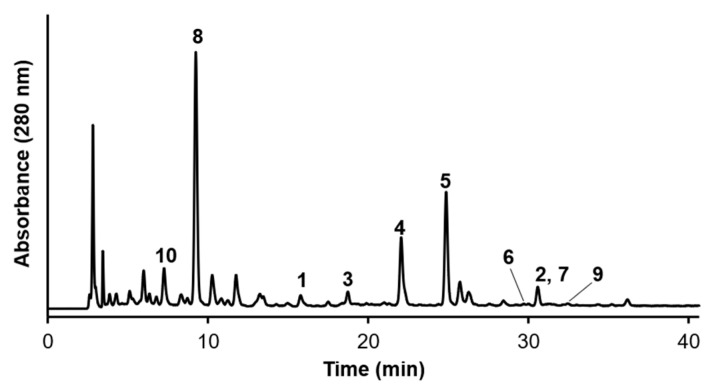
HPLC chromatogram of the ethanol extract of *C. officinalis* leaves; quercetin 3-*O*-β-glucoside (**1**), isorhamnetin 3-*O*-β-glucoside (**2**), quercetin 3-*O*-β-neohesperidoside (**3**), quercetin 3-*O*-(2″-*O*-α-rhamnosyl-6″-*O*-malonyl)-β-glucoside (**4**), quercetin 3-*O*-(6″-*O*-malonyl)-β-glucoside (**5**), quercetin 3-*O*-6″-*O*-methylmalonyl)-β-glucoside (**6**), isorhamnetin 3-*O*-(6″-*O*-malonyl)-β-glucoside (**7**), chlorogenic acid (**8**), 3,4-dicaffeoylquinic acid (**9**), and syringic acid (**10**).

**Figure 4:**
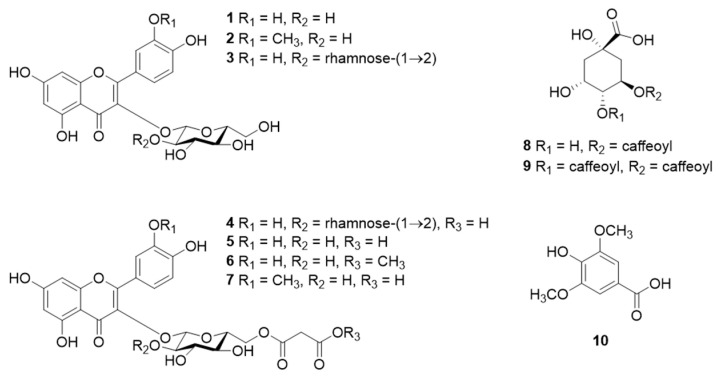
Structures of **1**–**10**.

The major components of *Calendula* leaves were determined by isolating ten known compounds using various chromatography techniques (Figure 3 and Figure 4). The compounds were identified by NMR and MS to be quercetin 3-*O*-β-glucoside (**1**) [18], isorhamnetin 3-*O*-β-glucoside (**2**) [18], quercetin 3-*O*-β-neohesperidoside (**3**) [19], quercetin 3-*O*-(2″-*O*-α-rhamnosyl-6″-*O*-malonyl)-β-glucoside (**4**) [19], quercetin 3-*O*-(6″-*O*-malonyl)-β-glucoside (**5**) [18], quercetin 3-*O*-6″-*O*-methylmalonyl)-β-glucoside (**6**) [18], isorhamnetin 3-*O*-(6″-*O*-malonyl)-β-glucoside (**7**) [20], chlorogenic acid (**8**) [21], 3,4-dicaffeoylquinic acid (**9**) [22], and syringic acid (**10**) [23]. NMR and MS spectra of **1**–**10** were indicated in Figures S1–S46. Compounds **1**–**3** and **8**–**10** were previously isolated from *C. officinalis* petals [24] but the present study is the first to the finding of **4**–**7** from *Calendula*.

### 2.3. COMT Inhibitory Activities of ***1***–***10***

We evaluated the COMT inhibitory activities of **1**–**10** (Table 1). All compounds except **7** and **10** exhibited high inhibitory activities, with IC_50_ values ≤100 µM. Compounds possessing quercetin skeletons as aglycones (**1** and **4**–**6**) showed high activity.

Quercetin is a remarkable natural COMT inhibitor [25,26], suggesting that quercetin glycosides and quercetin malonylated glycosides exhibit high activity. 3,4-Dicaffeoylquinic acid (**9**) exhibited the highest inhibitory activity toward COMT of the compounds isolated here and all active compounds possessed the catechol moiety. Previous studies reported that catecholic compounds inhibit COMT by interacting with the catechol substrate binding site [25,27], suggesting that COMT inhibitors from *C. officinalis* inhibit COMT competitively.

Tolcapone, entacapone, and opicapone are COMT inhibitors used to treat Parkinson’s disease [17]. However, tolcapone exhibits severe hepatotoxicity [28], and the toxicity of opicapone has not been evaluated in detail [29]. Therefore, COMT inhibitors with low toxicity and good safety profiles are required. Natural sources of pharmaceuticals could offer many potentially safe COMT inhibitors because of their low toxicity, and therefore *Calendula* leaves might be a source of safe and effective ingredients for the prevention and treatment of Parkinson’s disease.

## 3. Materials and Methods

### 3.1. General Experimental Procedures

One-dimensional and two-dimensional NMR spectra were acquired on an AVANCE III (400 MHz) (Bruker BioSpin, Rheinstetten, Germany), with chemical shifts expressed in ppm. The NMR spectra were referenced to residual solvent peaks (CD_3_OD: ^1^H NMR 3.30 ppm, ^13^C NMR 49.0 ppm). HR-ESI-MS spectra were acquired on a Thermo Fisher Scientific Q-Exactive HR-ESI-Orbitrap-MS (Waltham, MA, USA). Medium pressure liquid chromatography (MPLC) was conducted using an AI-580 system equipped with an ULTRA PAK ODS-SM-50D (50 µm, 50 × 300 mm, Yamazen Corporation, Osaka, Japan). Reversed-phase (RP)-HPLC separations were performed with a recycling system comprising a PU-2086 Plus Intelligent prep pump (Jasco, Tokyo, Japan), UV-2075 detector (Jasco, Tokyo, Japan), Capcell Pak UG120 C18 column (5 μm, 20 × 250 mm, Osaka Soda, Osaka, Japan), Capcell Pak UG120 C18 column (5 μm, 10 × 250 mm, Osaka Soda, Osaka, Japan), and HPLC-grade solvents. For analytical HPLC, a PU-4180 RHPLC pump (Jasco, Tokyo, Japan), MD-4017 photodiode array detector (Jasco, Tokyo, Japan), and AS-4050 HPLC autosampler (Jasco, Tokyo, Japan) were used. Data were analysed using ChromNAV software v.2 (Jasco, Tokyo, Japan).

### 3.2. Biological Material

*C. officinalis* leaves were collected in Hokkaido, Japan, in October 2019 and July 2020. The voucher numbers of each sample are 201910 and 202007, respectively.

### 3.3. Folin–Ciocalteu Colorimetric Method

The sample solution (150–1200 µg/mL *Calendula* samples (petal, leaf, and stem) and 10% Folin–Ciocalteu reagent) were preincubated at room temperature for 3 min, and then 10% Na_2_CO_3_ was added to the sample solution. After incubation at room temperature for 1 h, an aliquot was analyzed at wavelength 765 nm using a FlexStation^®^ 3 (Molecular Devices, San Jose, CA, USA). The total polyphenol contents were calculated as gallic acid equivalent.

### 3.4. Extraction and Isolation of Compounds in Calendula Leaves

Dried powdered *Calendula* leaves (Lot No. 201910, 20 g) were extracted with 70% ethanol (200 mL) under stirring at room temperature overnight, then the solids were removed by filtration. The filtrate was concentrated at reduced pressure to give the ethanol extracts (5.2 g). This extracts were suspended in H_2_O (150 mL) and partitioned successively with ethyl acetate (325 mL) to yield the H_2_O-1 fraction (3.7 g). An aliquot of this fraction (2.6 g) was subjected to MPLC with H_2_O–acetonitrile (90:10 (0 min); 60:40 (150 min); 0:100 (155 min); 0.1% trifluoroacetic acid (TFA)) to yield nine fractions (H_2_O-1 fr. 1, 1.5 g; H_2_O-1 fr. 2, 143 mg; H_2_O-1 fr. 3, 42 mg; H_2_O-1 fr. 4, 59 mg; H_2_O-1 fr. 5, 70 mg; H_2_O-1 fr. 6, 277 mg; H_2_O-1 fr. 7, 241 mg; H_2_O-1 fr. 8, 298 mg; H_2_O-1 fr. 9, 787 mg). H_2_O-1 fr. 2 was subjected to preparative RP-HPLC with H_2_O−acetonitrile (88:12, 0.1% TFA) as the eluent to give **8** (31 mg). H_2_O-1 fr. 3 was subjected to preparative RP-HPLC with H_2_O−acetonitrile (82:18, 0.1% TFA) as the eluent to give **3** (1.8 mg). H_2_O-1 fr. 4 was subjected to preparative RP-HPLC with H_2_O−acetonitrile (80:20, 0.1% TFA) as the eluent to give **4** (22 mg). H_2_O-1 fr. 5 was subjected to preparative RP-HPLC with H_2_O−acetonitrile (75:25, 0.1% TFA) as the eluent to give **5** (28 mg). 

A second sample of *Calendula* leaves (Lot No. 202007, 90 g) was extracted with methanol (2 L) under stirring at room temperature overnight, then the solids were removed by filtration. The filtrate was concentrated at reduced pressure to give the methanol extracts (25 g). This extracts were suspended in H_2_O (400 mL) and successively partitioned with *n*-hexane (800 mL) and ethyl acetate (600 mL) to give ethyl acetate (EA) (1.7 g) and H_2_O-2 fractions (15 g), respectively. The EA fraction (1.7 g) was subjected to silica gel column chromatography (50 × 280 mm), with *n*-hexane/ethyl acetate−methanol gradient mixtures (7:3, 400 mL; 3:2, 500 mL; 1:1, 300 mL; 2:3, 500 mL; 3:7, 600 mL; 1:4, 500 mL; 1:9, 700 mL; 0:1, 500 mL; MeOH 1 L) as eluents, to yield 12 fractions (EA fr. 1, 167 mg; EA fr. 2, 35 mg; EA fr. 3, 146 mg; EA fr. 4, 41 mg; EA fr. 5, 16 mg; EA fr. 6, 95 mg; EA fr. 7, 18 mg; EA fr. 8, 62 mg; EA fr. 9, 79 mg; EA fr. 10, 15 mg; EA fr. 11, 41 mg; EA fr. 12, 434 mg). EA fr. 12 (434 mg) was subjected to MPLC with H_2_O–acetonitrile (90:10 (0 min); 60:40 (140 min); 0:100 (155 min); 0.1% acetic acid) to yield seven fractions (EA-12 fr. 1, not calculated; EA-12 fr. 2, 28 mg; EA-12 fr. 3, 86 mg; EA-12 fr. 4, 29 mg; EA-12 fr. 5, 17 mg; EA-12 fr. 6, 62 mg; EA-12 fr. 7, 62 mg). EA-12 fr. 1 was subjected to preparative RP-HPLC with H_2_O−acetonitrile (95:5, 0.1% TFA) as the eluent to give **10** (2.1 mg). EA-12 fr. 2 was subjected to preparative RP-HPLC with H_2_O−acetonitrile (85:15, 0.1% TFA) as the eluent to give **1** (5.4 mg). EA-12 fr. 3 was subjected to preparative RP-HPLC with H_2_O−acetonitrile (80:20, 0.1% TFA) as the eluent to give **2** (1.1 mg) and **9** (0.9 mg). The H_2_O-2 fraction (15 g) was subjected to MPLC with H_2_O–acetonitrile (90:10 (0 min); 60:40 (215 min); 0:100 (220 min); 0.1% trifluoroacetic acid (TFA)) to yield 13 fractions (H_2_O-2 fr. 1, 165 mg; H_2_O-2 fr. 2, 267 mg; H_2_O-2 fr. 3, 194 mg; H_2_O-2 fr. 4, 705 mg; H_2_O-2 fr. 5, 107 mg; H_2_O-2 fr. 6, 83 mg; H_2_O-2 fr. 7, 170 mg; H_2_O-2 fr. 8, 153 mg; H_2_O-2 fr. 9, 40 mg; H_2_O-2 fr. 10, 16 mg; H_2_O-2 fr. 11, 56 mg; H_2_O-2 fr. 12, 39 mg; H_2_O-2 fr. 13, 25 mg). H_2_O-2 fr. 10 was subjected to preparative RP-HPLC with H_2_O−acetonitrile (80:20, 0.1% TFA) as the eluent to give **6** (0.9 mg). H_2_O-2 fr. 13 was subjected to preparative RP-HPLC with H_2_O−acetonitrile (80:20, 0.1% TFA) as the eluent to give **7** (2.1 mg). All conditions for preparative MPLC separations were as follows; detection wavelength: 280 nm, column: ULTRA PAK ODS-SM-50D (50 µm, 50 × 300 mm, Yamazen Corporation, Osaka, Japan), flow rate: 45 mL/min, temperature: room temperature. All conditions for preparative RP-HPLC separations were as follows; detection wavelength: 280 nm, column: Capcell Pak UG120 C18 column (5 μm, 10 or 20 × 250 mm, Osaka Soda, Osaka, Japan), flow rate: 4.8 or 9.6 mL/min, injection volume: 500–1000 μL, temperature: room temperature. 

The structures of **1**–**10** were determined based on 1D and 2D NMR, HRMS, and comparisons with data from previous studies.

### 3.5. COMT Inhibitory Assays

3-(Benzo[*d*]thiazol-2-yl)-7,8-dihydroxy-2*H*-chromen-2-one (3-BTD) was prepared based on a previous report [30]. Recombinant human COMT samples were expressed following an earlier report [31]. *S*-Adenosylmethionine (SAM) was purchased from New England Biolabs, Inc. (Ipswich, MA, USA). Tolcapone was purchased from Tokyo Chemical Industry Co. (Tokyo, Japan) and used as a positive control for this assay. COMT inhibitory assays were performed by following a previously reported method [32]. The assay buffer (50 mM Tris-HCl (pH 7.5), 1.5 mM MgCl_2_, 1 µM COMT, 200 µM SAM, and 0–100 µM inhibitor) was transferred to a 96-well microtiter plate and preincubated at 37 °C for 10 min. 3-BTD was added to the buffer solution at a final concentration of 20 µM (total volume: 150 µL), and the reaction was started. After incubation at 37 °C for 4 min, 3% aqueous HClO_4_ (30 μL) was added to terminate the reaction. An aliquot was analysed using a FlexStation^®^ 3 (Molecular Devices, San Jose, CA, USA) to identity the product. 3-(Benzo[*d*]thiazol-2-yl)-7-hydroxy-8-methoxy-2*H*-chromen-2-one (3-BTMD), the methylated product, was excited at 390 nm, and the emission wavelength was 510 nm. The percentage inhibition was calculated according to the following equation: Inhibition (%) = [(fluorescence intensity in the control experiment) − (fluorescence intensity in the sample experiment)] × 100 / (fluorescence intensity in the control experiment).

## Figures and Tables

**Table 1:** COMT inhibitory activities (IC_50_) of **1**–**10**.

Compound	IC_50_ (µM)
**1**	50
**2**	55
**3**	71
**4**	65
**5**	59
**6**	42
**7**	>100
**8**	100
**9**	21
**10**	>100
Tolcapone	0.55

## Data Availability

The data presented in this study are available upon request from the corresponding author.

## Supplementary Materials

The following supporting information can be downloaded at: https://www.mdpi.com/article/10.3390/molecules28031333/s1, Figure S1. HR-ESI-MS spectrum of **1** (negative mode); Figure S2. ^1^H NMR spectrum of **1** in CD_3_OD (400 MHz); Figure S3. ^13^C NMR spectrum of **1** in CD_3_OD (100 MHz); Figure S4. HSQC spectrum of **1** in CD_3_OD; Figure S5. HMBC spectrum of **1** in CD_3_OD; Figure S6. HR-ESI-MS spectrum of **2** (positive mode); Figure S7. ^1^H NMR spectrum of **2** in CD_3_OD (400 MHz); Figure S8. ^13^C NMR spectrum of **2** in CD_3_OD (100 MHz); Figure S9. HSQC spectrum of **2** in CD_3_OD; Figure S10. HMBC spectrum of **2** in CD_3_OD; Figure S11. HR-ESI-MS spectrum of **3** (negative mode); Figure S12. ^1^H NMR spectrum of **3** in CD_3_OD (400 MHz); Figure S13. ^13^C NMR spectrum of **3** in CD_3_OD (100 MHz); Figure S14. ^13^C NMR spectrum of **3** in CD_3_OD (100 MHz); Figure S15. HMBC spectrum of **3** in CD_3_OD; Figure S16. HR-ESI-MS spectrum of **4** (negative mode); Figure S17. ^1^H NMR spectrum of **4** in CD_3_OD (400 MHz); Figure S18. ^13^C NMR spectrum of **4** in CD_3_OD (100 MHz); Figure S19. HSQC spectrum of **4** in CD_3_OD; Figure S20. HMBC spectrum of **4** in CD_3_OD; Figure S21. HR-ESI-MS spectrum of **5** (negative mode); Figure S22. ^1^H NMR spectrum of **5** in CD_3_OD (400 MHz); Figure S23. ^13^C NMR spectrum of **5** in CD_3_OD (100 MHz); Figure S24. HSQC spectrum of **5** in CD_3_OD; Figure S25. HMBC spectrum of **5** in CD_3_OD; Figure S26. HR-ESI-MS spectrum of **6** (positive mode); Figure S27. ^1^H NMR spectrum of **6** in CD_3_OD (400 MHz); Figure S28. ^13^C NMR spectrum of **6** in CD_3_OD (100 MHz); Figure S29. HSQC spectrum of **6** in CD_3_OD; Figure S30. HMBC spectrum of **6** in CD_3_OD; Figure S31. HR-ESI-MS spectrum of **7** (negative mode); Figure S32. ^1^H NMR spectrum of **7** in CD_3_OD (400 MHz); Figure S33. ^13^C NMR spectrum of **7** in CD_3_OD (100 MHz); Figure S34. HSQC spectrum of **7** in CD_3_OD; Figure S35. HMBC spectrum of **7** in CD_3_OD; Figure S36. HR-ESI-MS spectrum of **8** (negative mode); Figure S37. ^1^H NMR spectrum of **8** in CD_3_OD (400 MHz); Figure S38. ^13^C NMR spectrum of **8** in CD_3_OD (100 MHz); Figure S39. HR-ESI-MS spectrum of **9** (positive mode); Figure S40. ^1^H NMR spectra of **9** (**A**) and 3,4-dicaffeoylquinic acid (**B**) in CD_3_OD (400 MHz); Figure S41. HPLC chromatograms of **9** and 3,4-dicaffeoylquinic acid; Figure S42. HR-ESI-MS spectrum of **10** (negative mode); Figure S43. ^1^H NMR spectrum of **10** in CD_3_OD (400 MHz); Figure S44. ^13^C NMR spectrum of **10** in CD_3_OD (100 MHz); Figure S45. HSQC spectrum of **10** in CD_3_OD; Figure S46. HMBC spectrum of **10** in CD_3_OD.

## Sample Availability

Samples are available from the authors upon reasonable request.

## References

[1] Muley B.P., Khadabadi S.S., Banarase N.B. (2009). Phytochemical constituents and pharmacological activities of *Calendula officinalis* Linn (Asteraceae): A review. Trop. J. Pharm. Res..

[2] Arora D., Rani A., Sharma A. (2013). A review on phytochemistry and ethnopharmacological aspects of genus *Calendula*. Pharmacogn. Rev..

[3] Frankič T., Salobir K., Salobir J. (2009). The comparison of in vivo antigenotoxic and antioxidative capacity of two propylene glycol extracts of *Calendula officinalis* (marigold) and vitamin E in young growing pigs. J. Anim. Physiol. Anim. Nutr. (Berl.).

[4] Pires T.C.S.P., Dias M.I., Barros L., Calhelha R.C., Alves M.J., Oliveira M.B.P.P., Santos-Buelga C., Ferreira I.C.F.R. (2018). Edible flowers as sources of phenolic compounds with bioactive potential. Food Res. Int..

[5] Ukiya M., Akihisa T., Yasukawa K., Tokuda H., Suzuki T., Kimura Y. (2006). Anti-inflammatory, anti-tumor-promoting, and cytotoxic activities of constituents of marigold (*Calendula officinalis*) flowers. J. Nat. Prod..

[6] Akihisa T., Yasukawa K., Oinuma H., Kasahara Y., Yamanouchi S., Takido M., Kumaki K., Tamura T. (1996). Triterpene alcohols from the flowers of compositae and their anti-inflammatory effects. Phytochemistry.

[7] Yoshikawa M., Murakami T., Kishi A., Kageura T., Matsuda H. (2001). Medicinal flowers. III. marigold. (1): Hypoglycemic, gastric emptying inhibitory, and gastroprotective principles and new oleanane-Type triterpene oligoglycosides, calendasaponins A, B, C, and D, from Egyptian *Calendula officinalis*. Chem. Pharm. Bull..

[8] Śliwowski J., Dziewanowska K., Kasprzyk Z. (1973). Ursadiol: A new triterpene diol from *Calendula officinalis* flowers. Phytochemistry.

[9] Wilkomirski B., Kasprzyk Z. (1979). Free and ester-bound triterpene alcohols and sterols in cellular subfractions of *Calendula officinalis* flowers. Phytochemistry.

[10] Bakó E., Deli J., Tóth G. (2002). HPLC study on the carotenoid composition of *Calendula* products. J. Biochem. Biophys. Methods.

[11] Carvalho A.R., Diniz R.M., Suarez M.A.M., Figueiredo C.S.S.S., Zagmignan A., Grisotto M.A.G., Fernandes E.S., da Silva L.C.N. (2018). Use of some Asteraceae plants for the treatment of wounds: From ethnopharmacological studies to scientific evidences. Front. Pharmacol..

[12] Andersen F.A., Bergfeld W.F., Belsito D.V., Hill R.A., Klaassen C.D., Liebler D.C., Marks J.G., Shank R.C., Slaga T.J., Snyder P.W. (2010). Final report of the cosmetic ingredient review expert panel amended safety assessment of *Calendula officinalis*-derived cosmetic ingredients. Int. J. Toxicol..

[13] Szabo K., Mitrea L., Calinoiu L.F., Teleky B.E., Martau G.A., Plamada D., Pascuta M.S., Nemes S.A., Varvara R.A., Vodnar D.C. (2022). Natural polyphenol recovery from apple-, cereal-, and tomato-processing by-products and related health-promoting properties. Molecules.

[14] Leyva-López N., Lizárraga-Velázquez C.E., Hernández C., Sánchez-Gutiérrez E.Y. (2020). Exploitation of agro-industrial waste as potential source of bioactive compounds for aquaculture. Foods.

[15] Tunbridge E.M., Harrison P.J., Weinberger D.R. (2006). Catechol-*o*-methyltransferase, cognition, and psychosis: Val158Met and beyond. Biol. Psychiatry.

[16] Bilder R.M., Volavka J., Lachman H.M., Grace A.A. (2004). The catechol-*O*-methyltransferase polymorphism: Relations to the tonic-phasic dopamine hypothesis and neuropsychiatric phenotypes. Neuropsychopharmacology.

[17] Silva T.B., Borges F., Serrão M.P., Soares-da-Silva P. (2020). Liver says no: The ongoing search for safe catechol *O*-methyltransferase inhibitors to replace tolcapone. Drug Discov. Today.

[18] Jaramillo K., Dawid C., Hofmann T., Fujimoto Y., Osorio C. (2011). Identification of antioxidative flavonols and anthocyanins in *Sicana odorifera* fruit peel. J. Agric. Food Chem..

[19] Kazuma K., Noda N., Suzuki M. (2003). Malonylated flavonol glycosides from the petals of *Clitoria ternatea*. Phytochemistry.

[20] Wald B., Wray V., Galensa R., Herrmann K. (1989). Malonated flavonol glycosides and 3,5-dicaffeoylquinic acid from pears. Phytochemistry.

[21] Jin H.G., Kim A.R., Ko H.J., Woo E.R. (2015). A new megastigmane glycoside from *Akebia quinata*. Arch. Pharm. Res..

[22] Kim J.Y., Cho J.Y., Ma Y.K., Park K.Y., Lee S.H., Ham K.S., Lee H.J., Park K.H., Moon J.H. (2011). Dicaffeoylquinic acid derivatives and flavonoid glucosides from glasswort (*Salicornia herbacea* L.) and their antioxidative activity. Food Chem..

[23] Xiao C., Dai H., Liu H., Wang Y., Tang H. (2008). Revealing the metabonomic variation of rosemary extracts using ^1^H NMR spectroscopy and multivariate data analysis. J. Agric. Food Chem..

[24] Olennikov D.N., Kashchenko N.I. (2013). New Isorhamnetin glycosides and other phenolic compounds from *Calendula officinalis*. Chem. Nat. Compd..

[25] Cao Y., Chen Z.-J., Jiang H.-D., Chen J.-Z. (2014). Computational studies of the regioselectivities of COMT-catalyzed *Meta*-/*Para*-O methylations of luteolin and quercetin. J. Phys. Chem. B.

[26] Zhao D.-F., Fan Y.-F., Yu H.-N., Hou F.-B., Xiang Y.-W., Wang P., Ge G.-B., Yang L., Xu J.-G. (2021). Discovery and characterization of flavonoids in vine tea as catechol-*O*-methyltransferase inhibitors. Fitoterapia.

[27] Ikeda M., Iijima H., Shinoda I., Iwamoto H., Takeda Y. (2017). Inhibitory effect of bovine lactoferrin on catechol-*O*-methyltransferase. Molecules.

[28] Olanow C.W. (2000). Tolcapone and hepatotoxic effects. Arch. Neurol..

[29] Fabbri M., Ferreira J.J., Lees A., Stocchi F., Poewe W., Tolosa E., Rascol O. (2018). Opicapone for the treatment of Parkinson’s disease: A review of a new licensed medicine. Mov. Disord..

[30] Wang P., Xia Y.-L., Zou L.-W., Qian X.-K., Dou T.-Y., Jin Q., Li S.-Y., Yu Y., Wang D.-D., Luo Q. (2017). An optimized two-photon fluorescent probe for biological sensing and imaging of catechol-*O*-methyltransferase. Chem. Eur. J..

[31] Miyata R., Motoyama T., Nakano S., Ito S., Mukaide K., Vongsak B., Kumazawa S. (2021). Catechol-*O*-methyltransferase inhibitors isolated from Thai propolis. Nat. Prod. Commun..

[32] Miyata R., Hoshino S., Ahn M.R., Kumazawa S. (2022). Chemical profiles of Korean bee pollens and their catechol-*O*-methyltransferase inhibitory activities. J. Agric. Food Chem..

