# Supplementary material for: Catechol-O-methyltransferase Inhibitors from Calendula officinalis Leaf
Source: Molecules. 2023 Jan 30;28(3):1333. doi: 10.3390/molecules28031333 (PMC9919311; doi:10.3390/molecules28031333)
Supplement: Supplementary file 1 [file molecules-28-01333-s001.zip › molecules-2176588-supplementary.pdf]

Supplementary Materials

Catechol-*O*-methyltransferase Inhibitors from *Calendula officinalis* Leaf

Wataru Kadowaki [1,†], Ryo Miyata [1,†], Misa Fujinami [2], Yoshizumi Sato [2] and Shigenori Kumazawa [1,*]

[1]Graduate School of Integrated Pharmaceutical and Nutritional Sciences, University of Shizuoka, Shizuoka, Japan
[2]Kisho Corporation, Tokyo, Japan

[*]Correspondence: kumazawa@u-shizuoka-ken.ac.jp
[†] These authors contributed equally to this work.

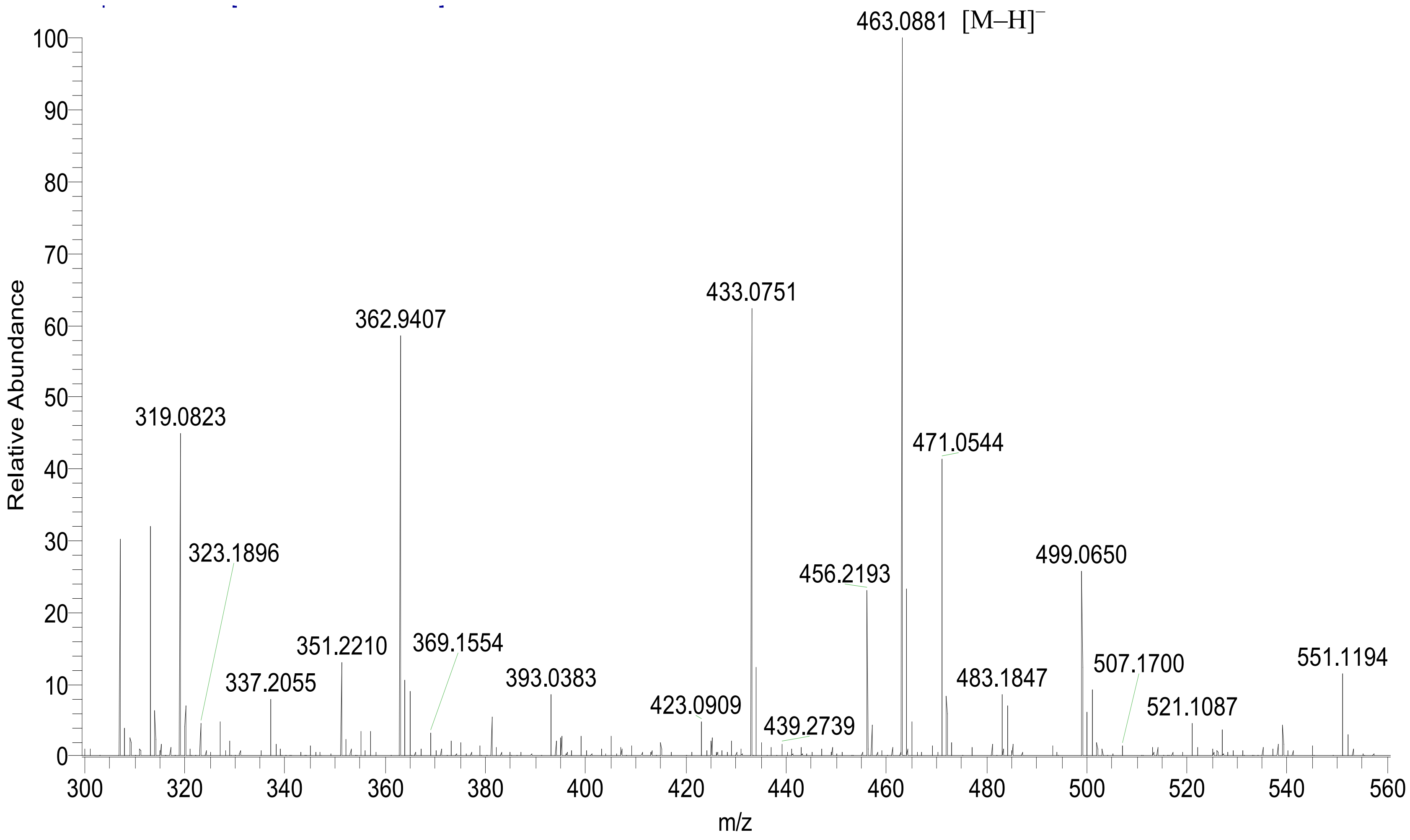

Figure S1. HRESIMS spectrum of **1** (negative mode).

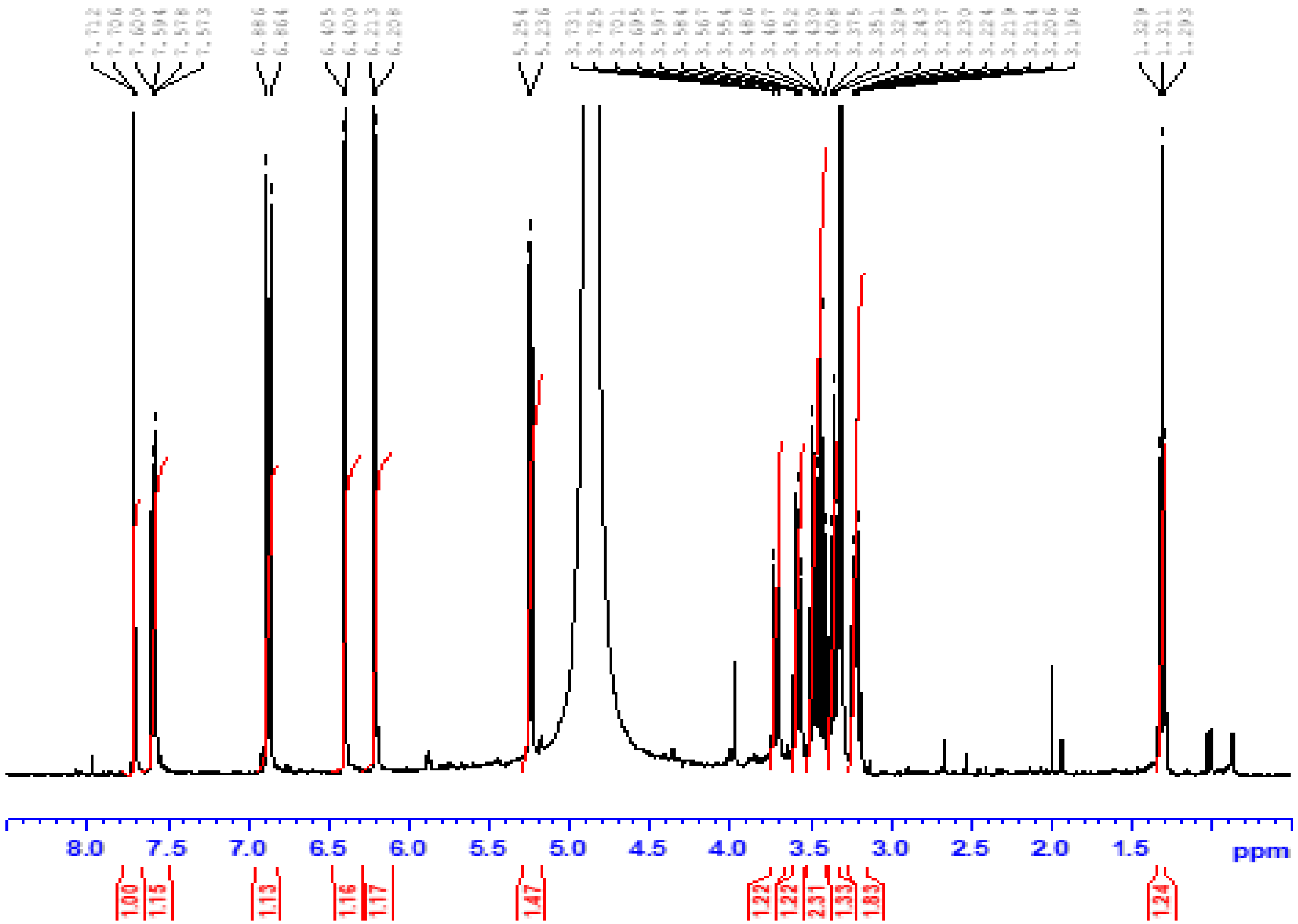

Figure S2. ^{1}H NMR spectrum of **1** in CD_3OD (400 MHz).

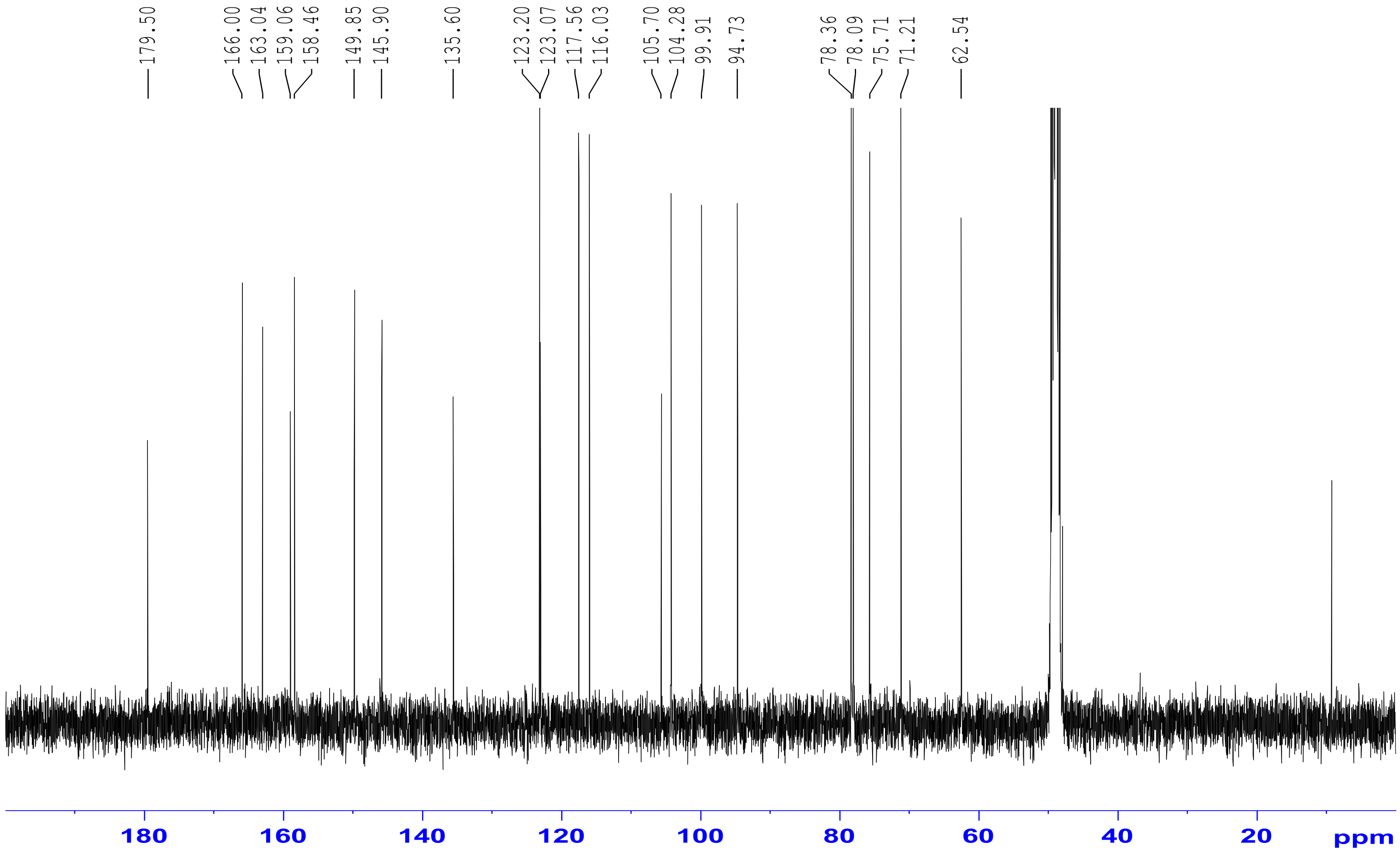

Figure S3. ^{13}C NMR spectrum of **1** in CD_3OD (100 MHz).

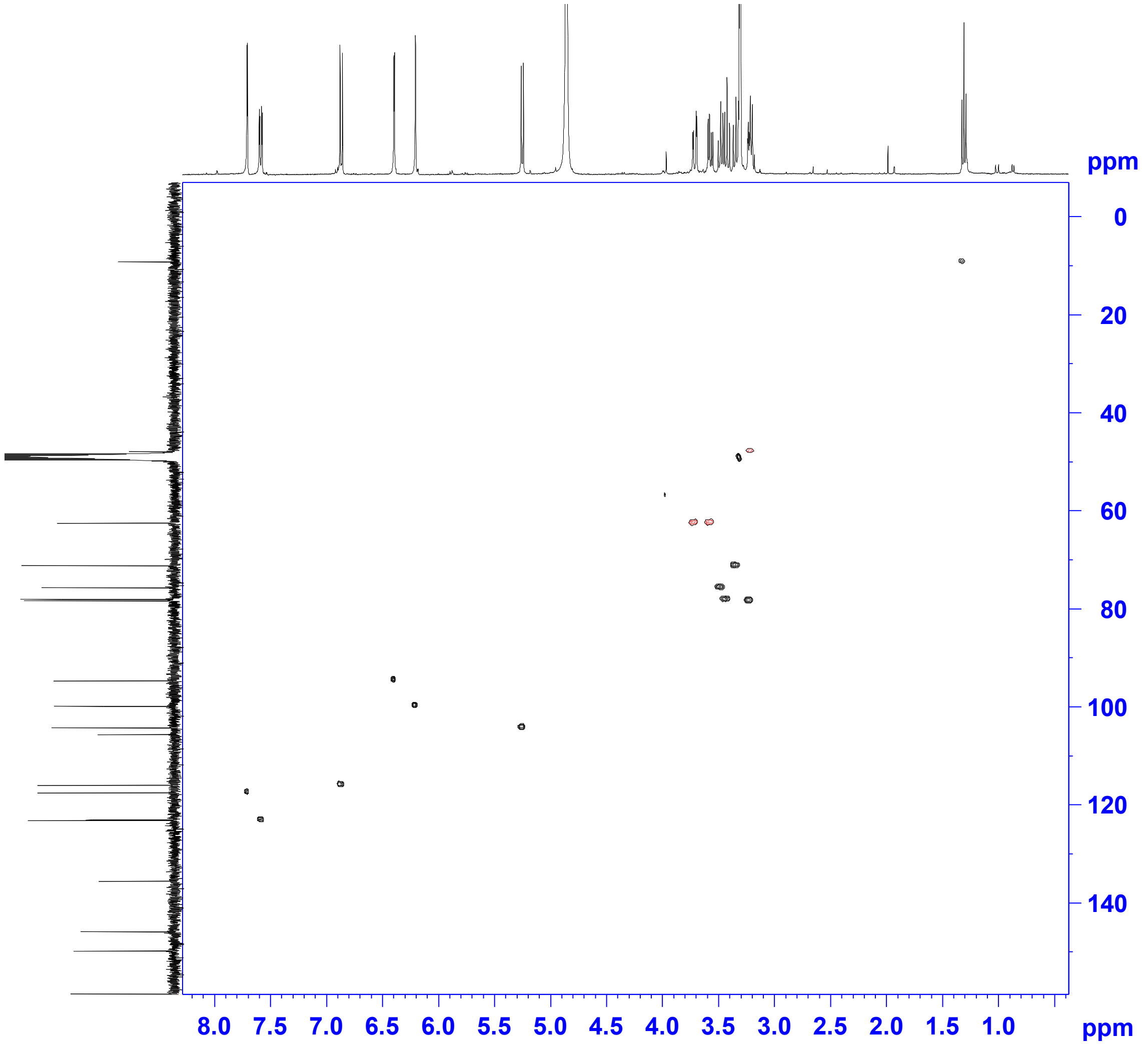

Figure S4. HSQC spectrum of **1** in CD_3OD.

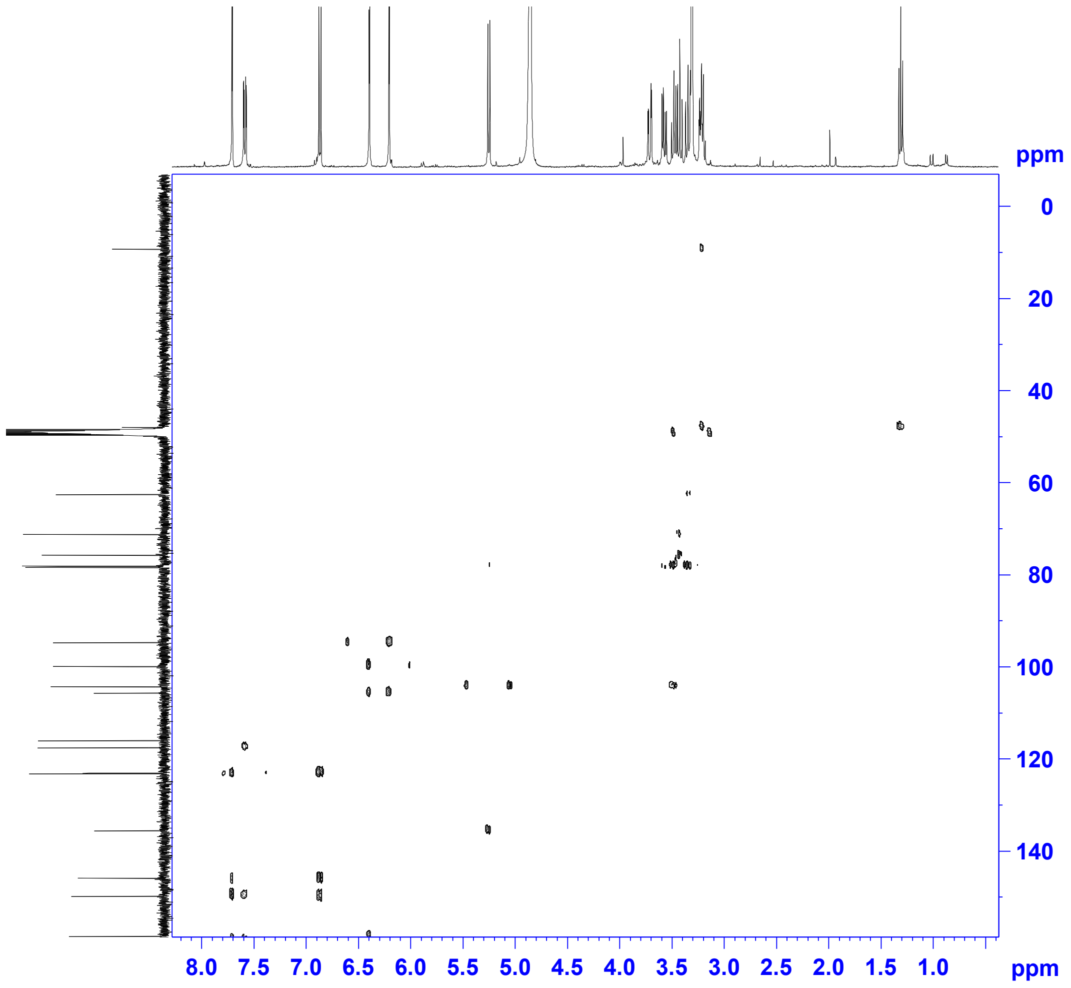

Figure S5. HMBC spectrum of **1** in CD_3OD.

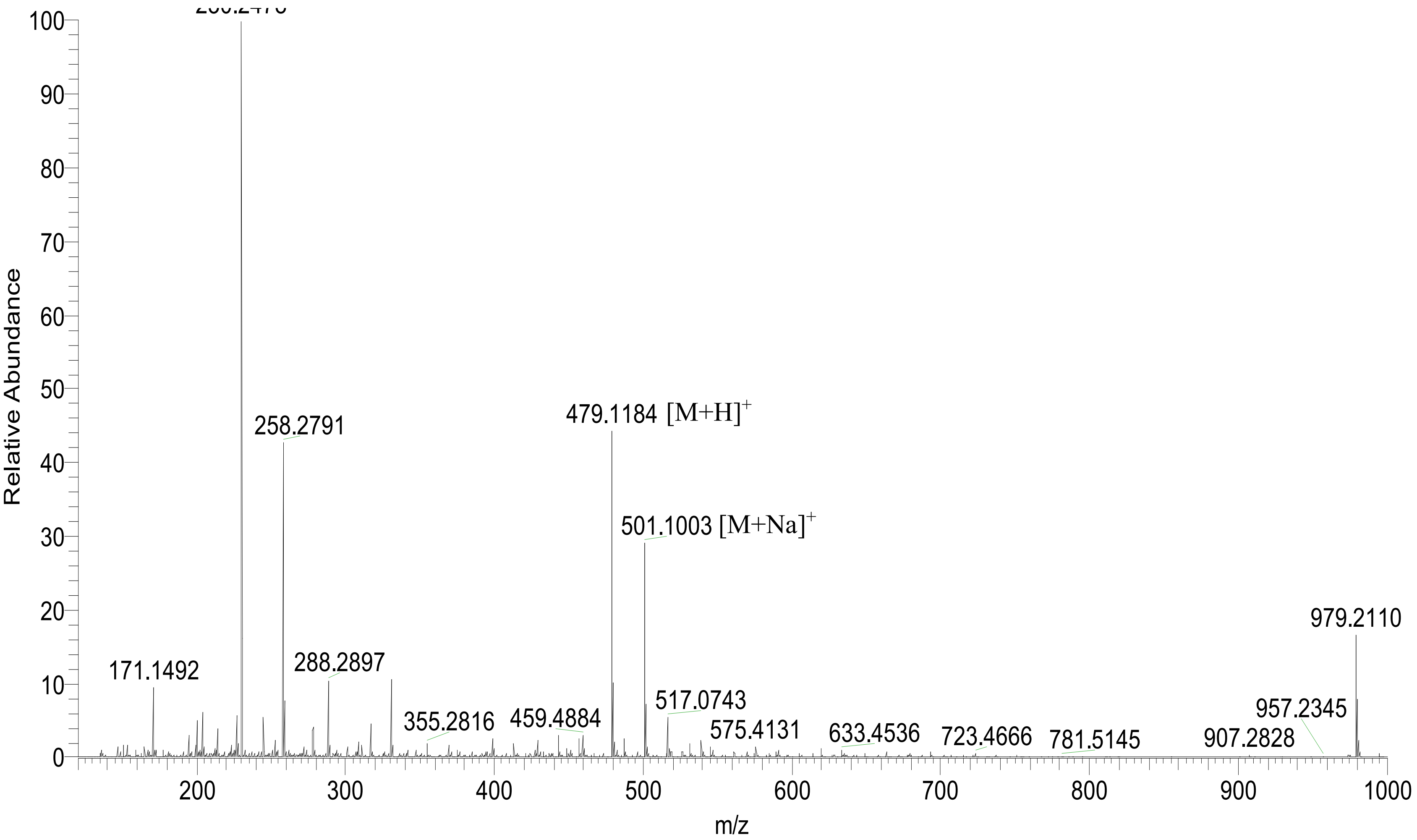

Figure S6. HRESIMS spectrum of **2** (positive mode).

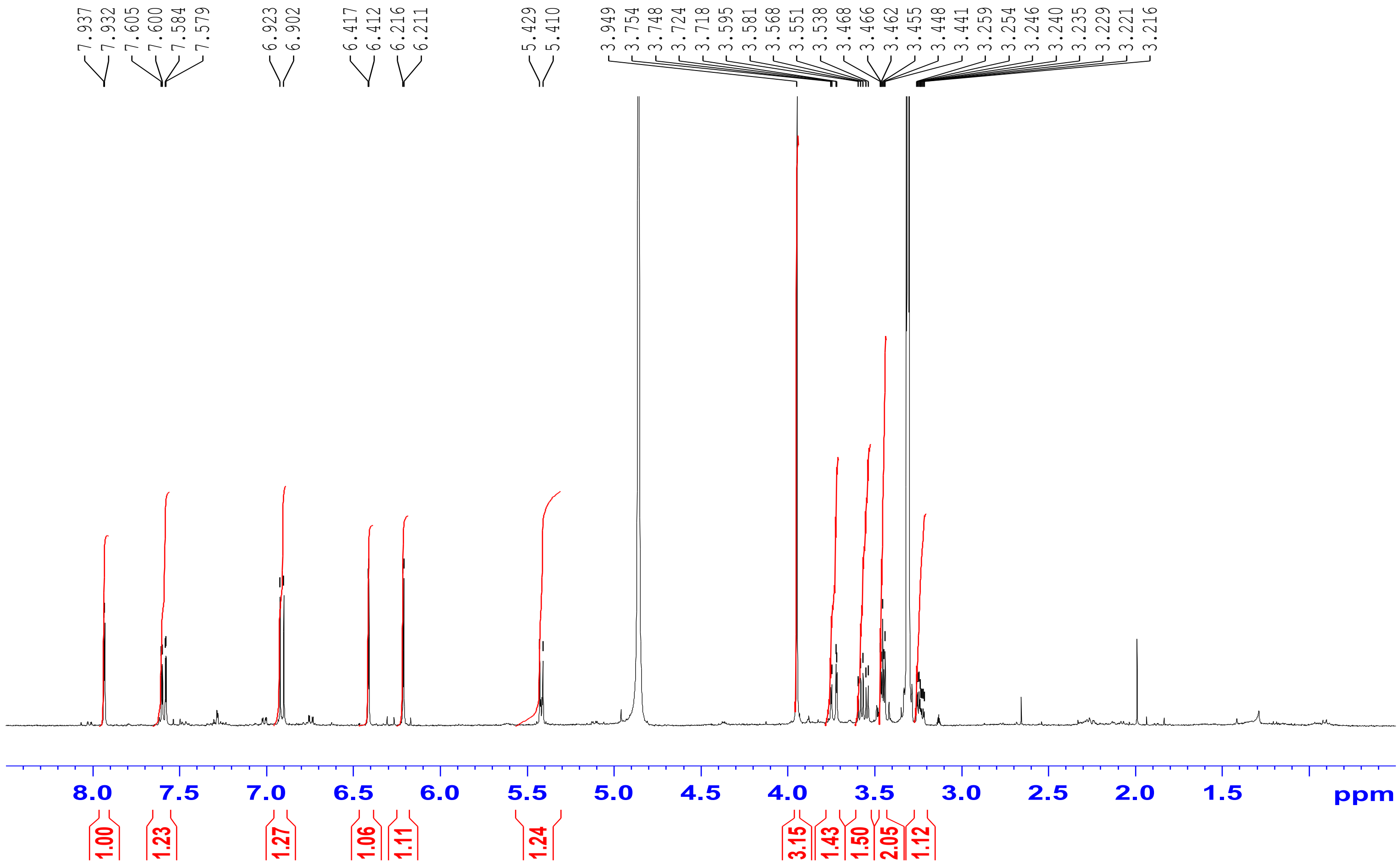

Figure S7. ^{1}H NMR spectrum of **2** in CD_3OD (400 MHz).

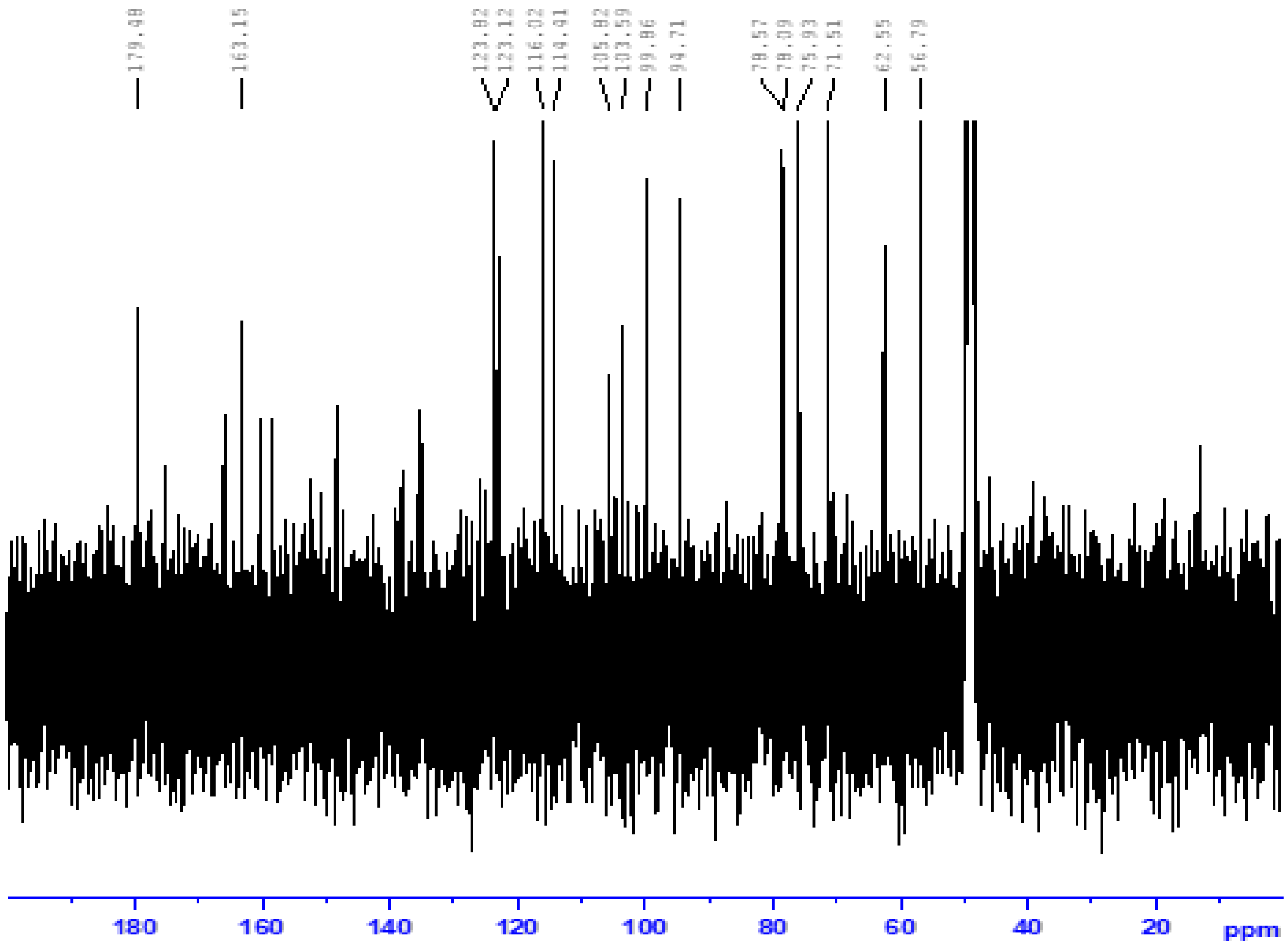

Figure S8. ^{13}C NMR spectrum of **2** in CD_3OD (100 MHz).

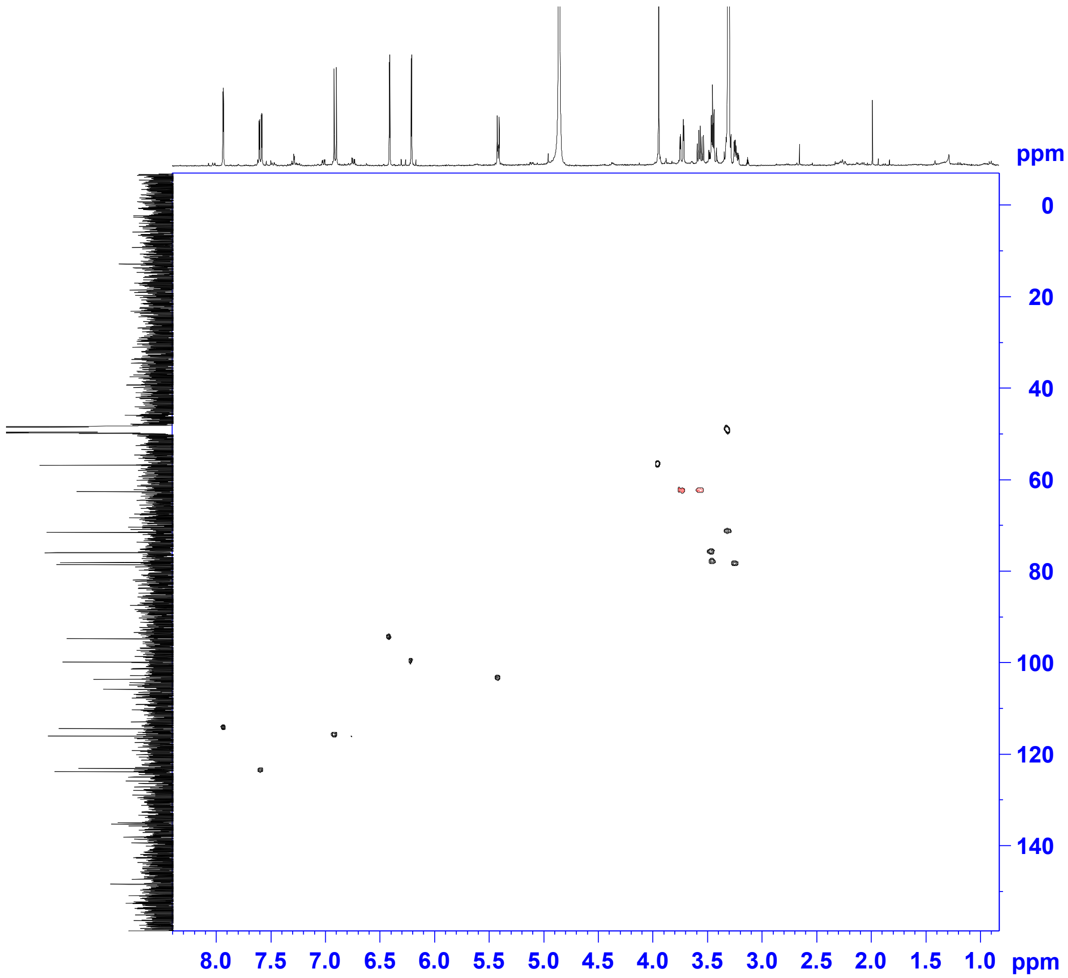

Figure S9. HSQC spectrum of **2** in CD_3OD.

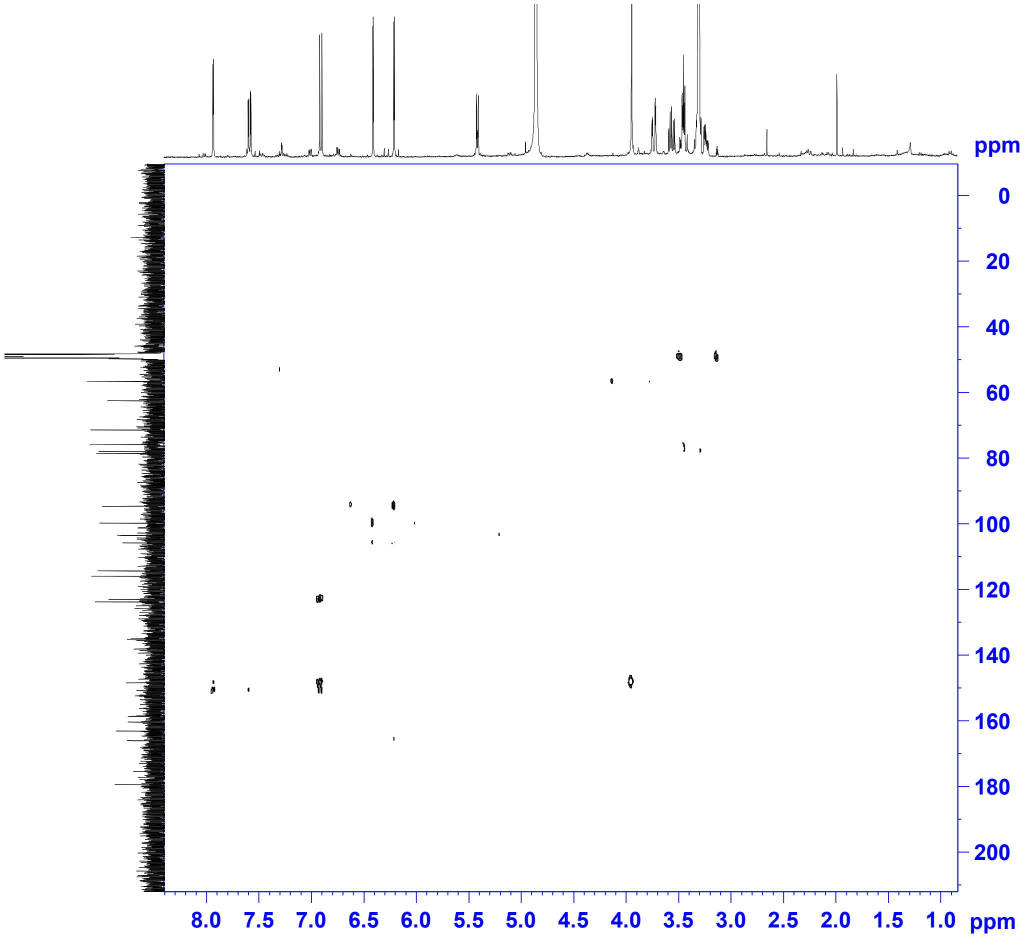

Figure S10. HMBC spectrum of **2** in CD_3OD.

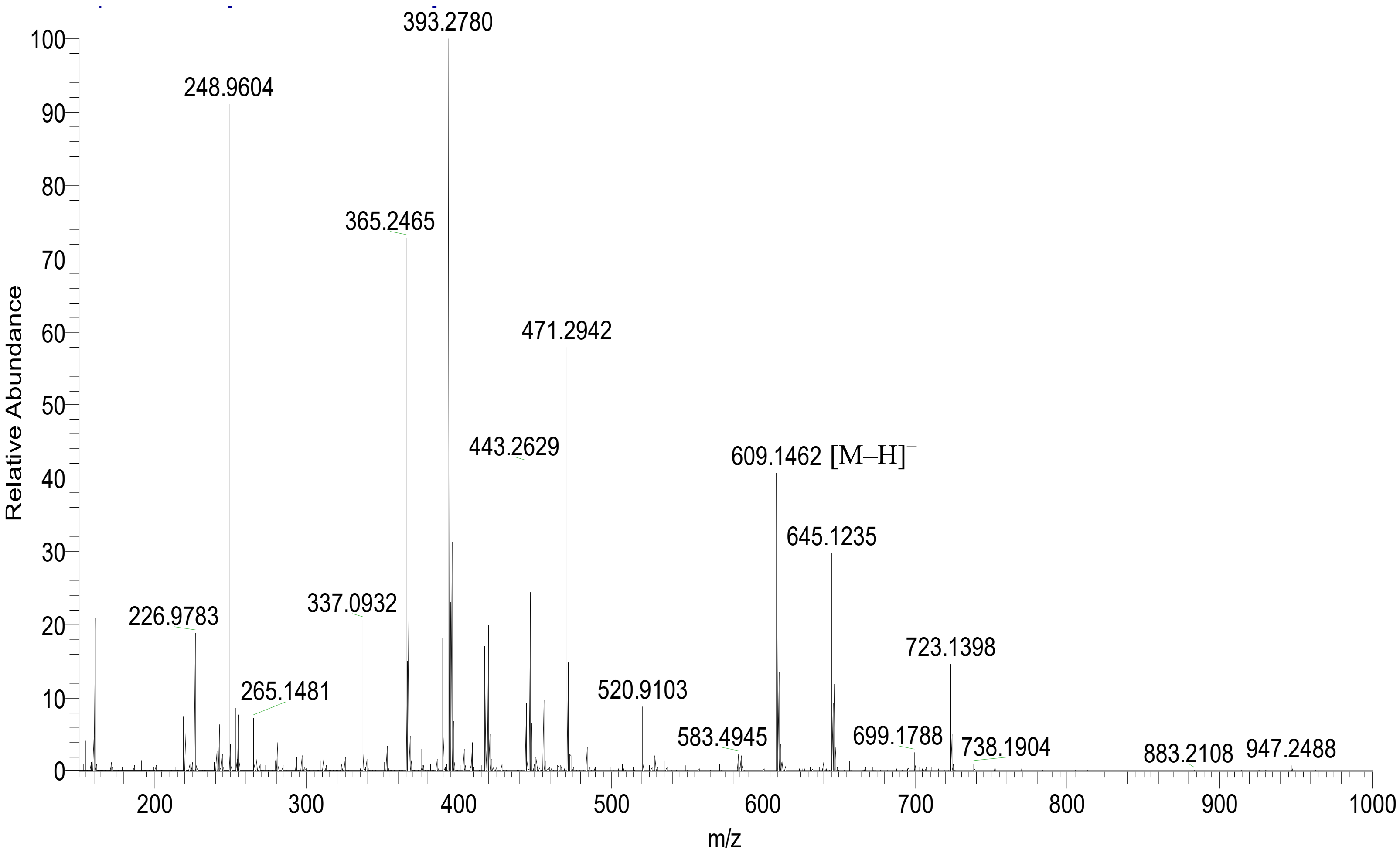

Figure S11. HRESIMS spectrum of **3** (negative mode).

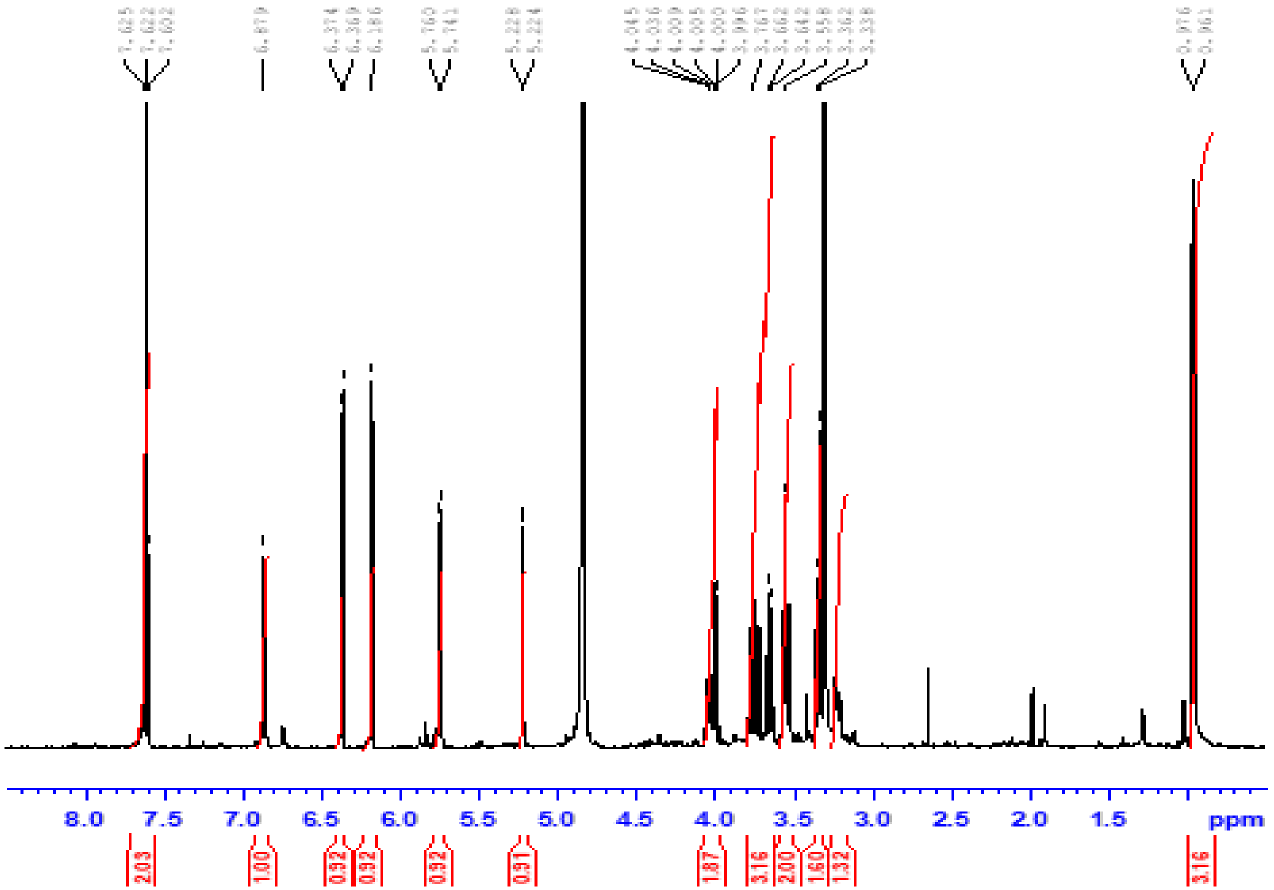

Figure S12. ^{1}H NMR spectrum of **3** in CD_3OD (400 MHz).

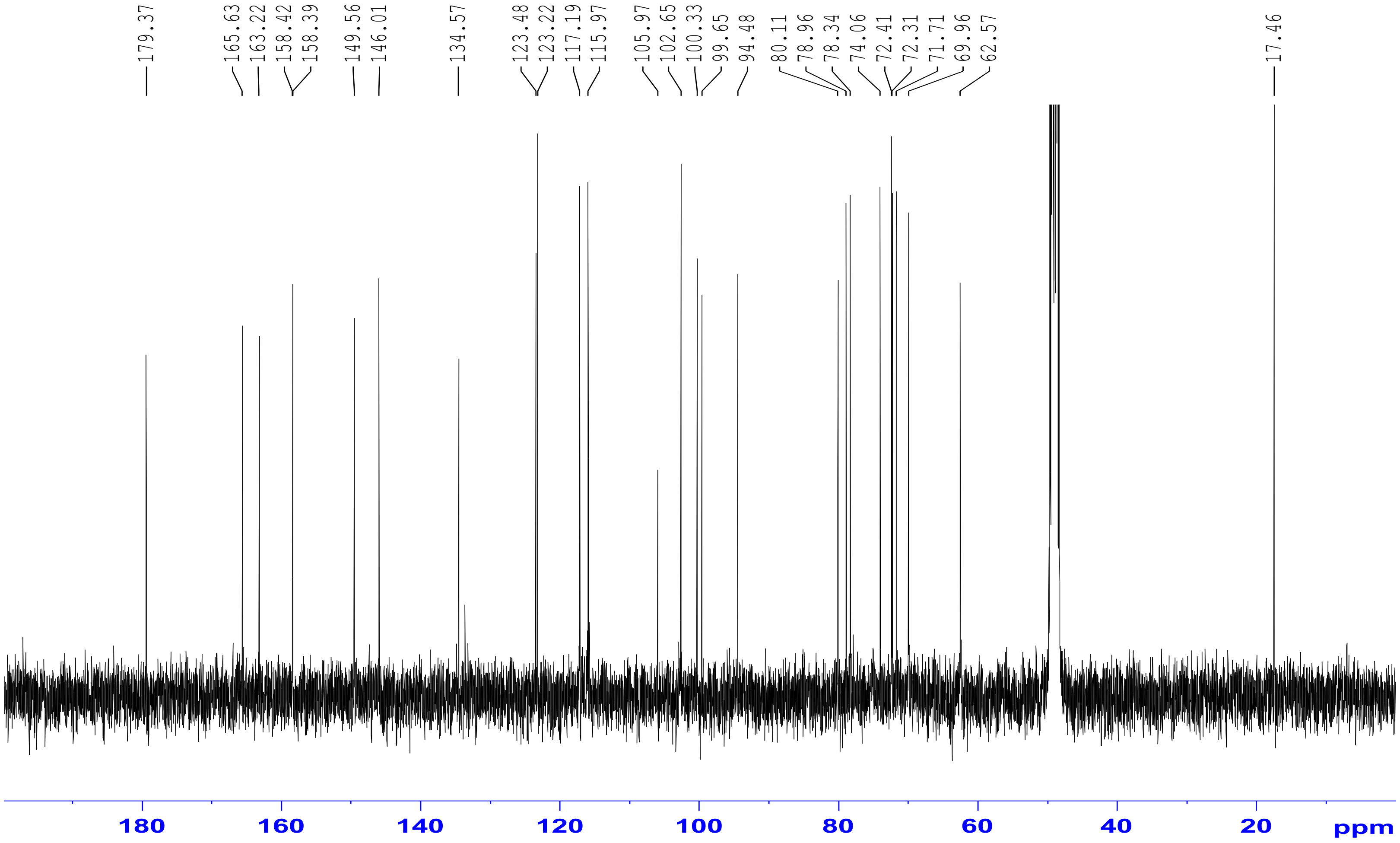

Figure S13. ^{13}C NMR spectrum of **3** in CD_3OD (100 MHz).

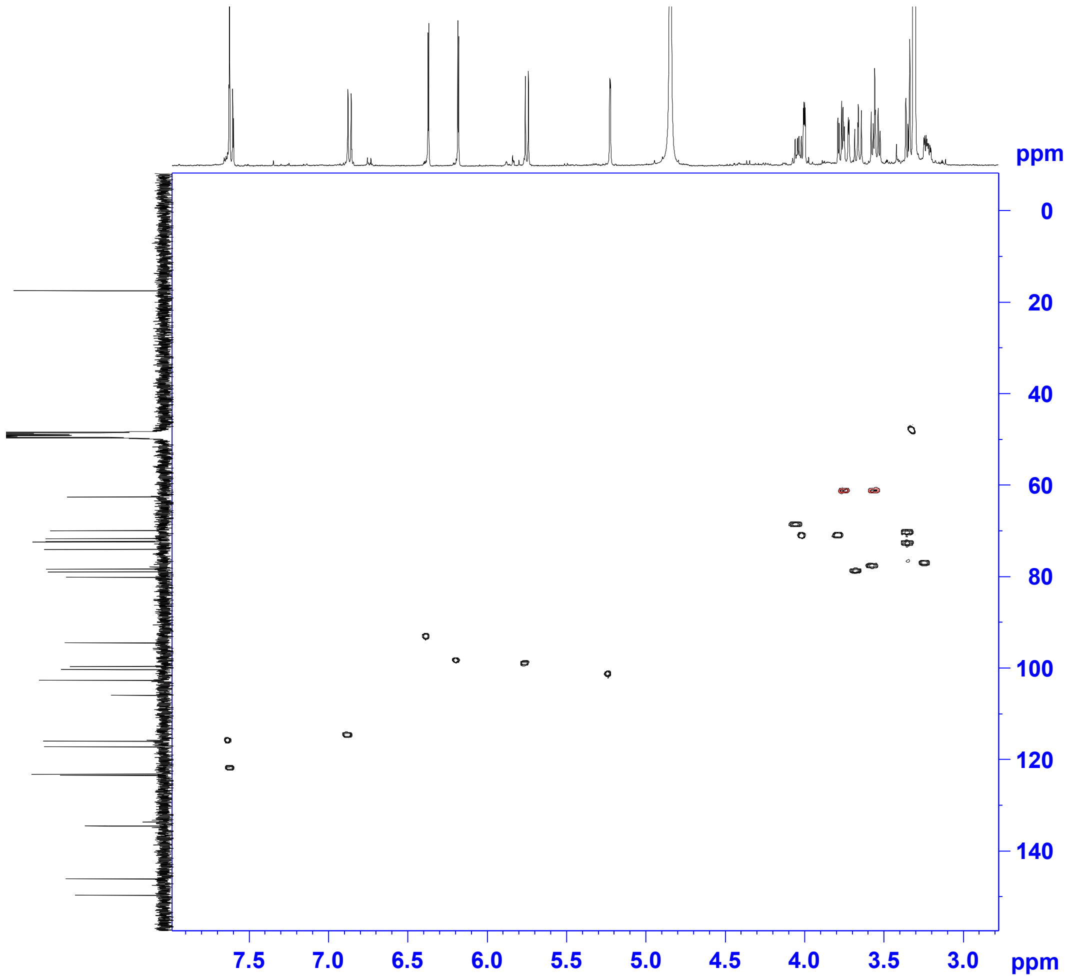

Figure S14. HSQC spectrum of **3** in CD_3OD.

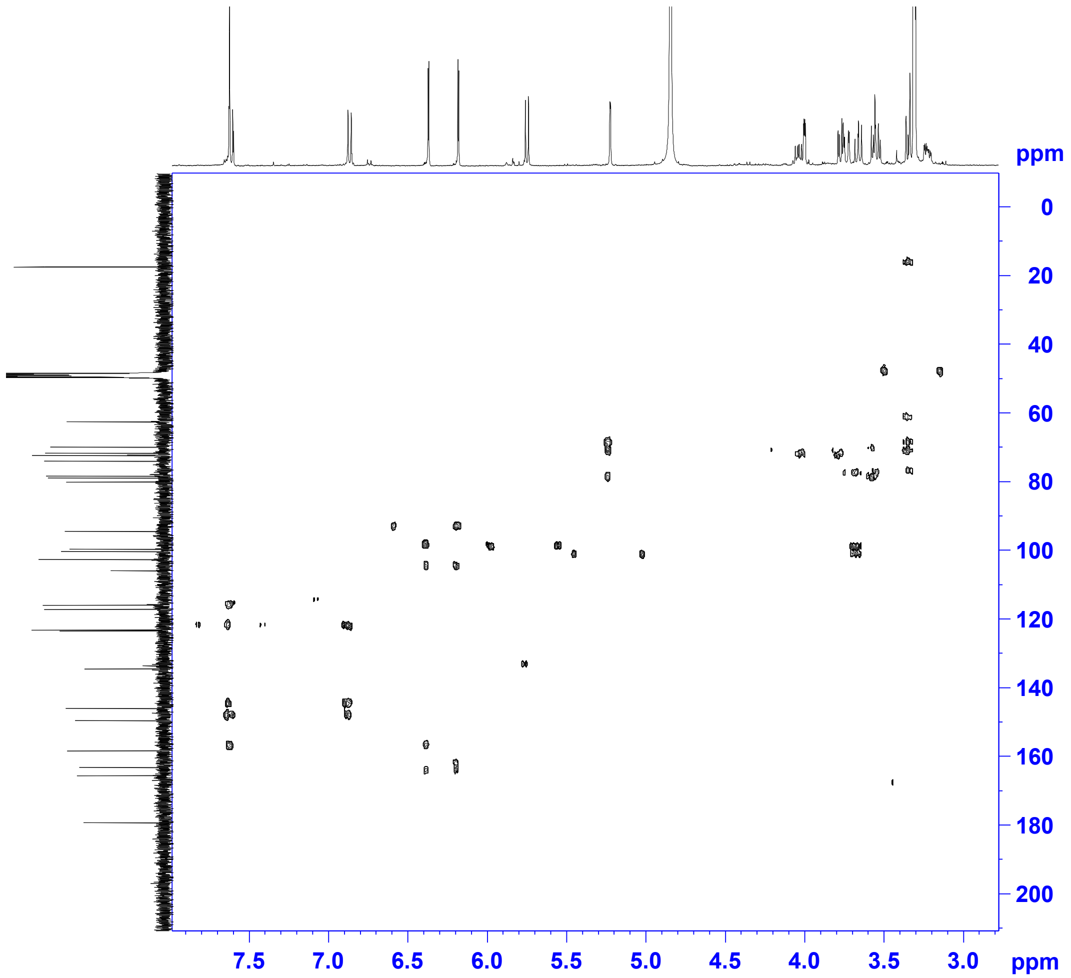

Figure S15. HMBC spectrum of **3** in CD_3OD.

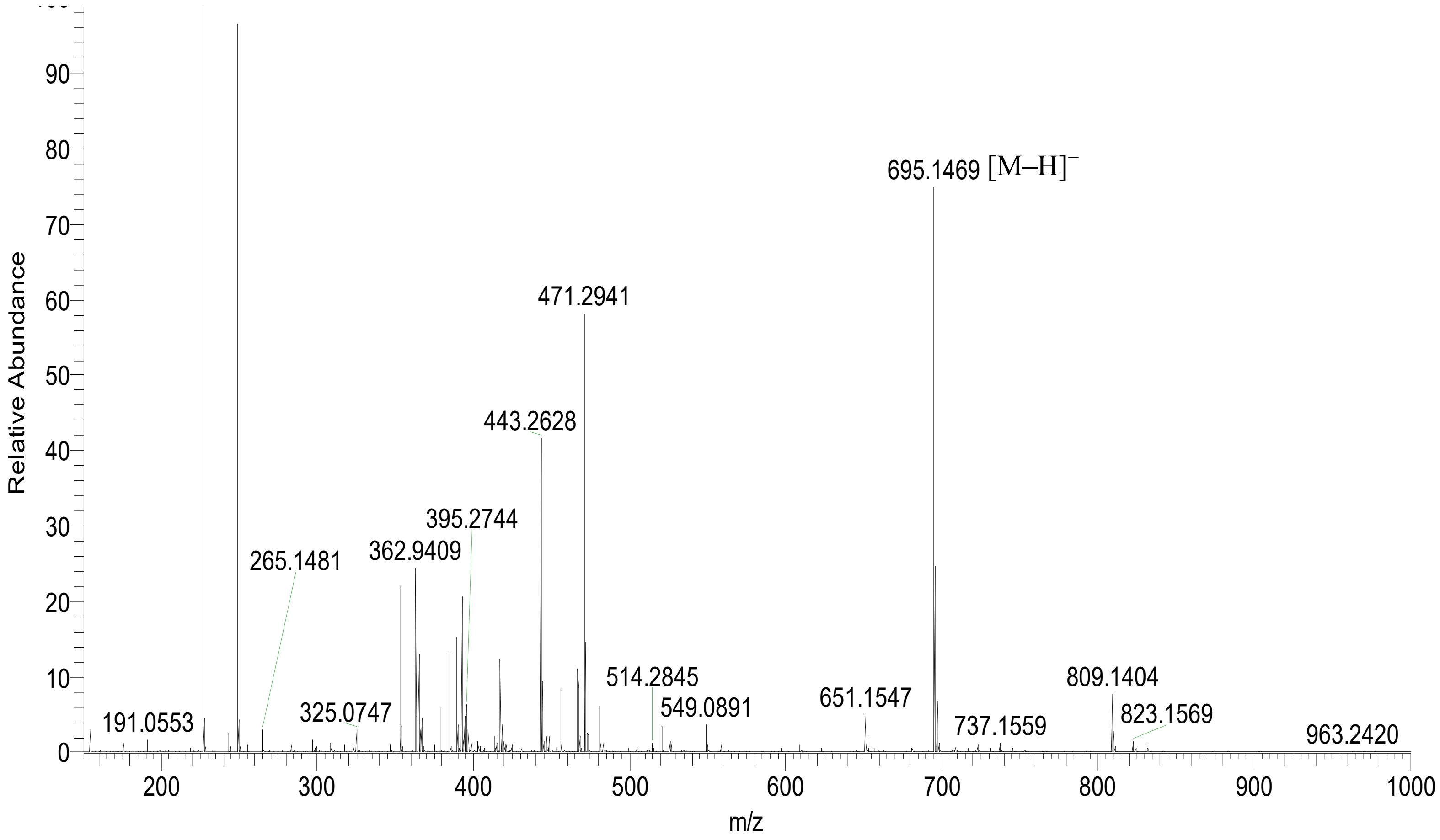

Figure S16. HRESIMS spectrum of **4** (negative mode).

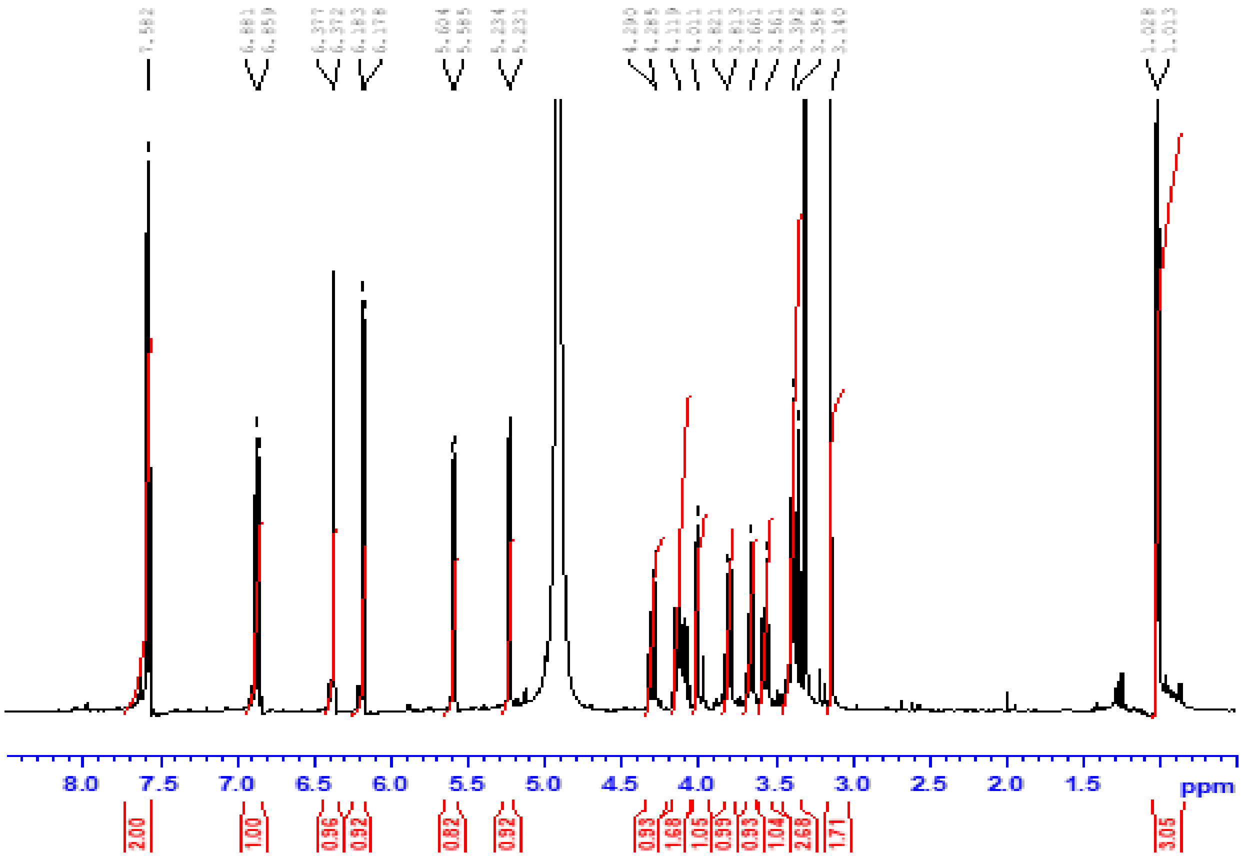

Figure S17. ^{1}H NMR spectrum of **4** in CD_3OD (400 MHz).

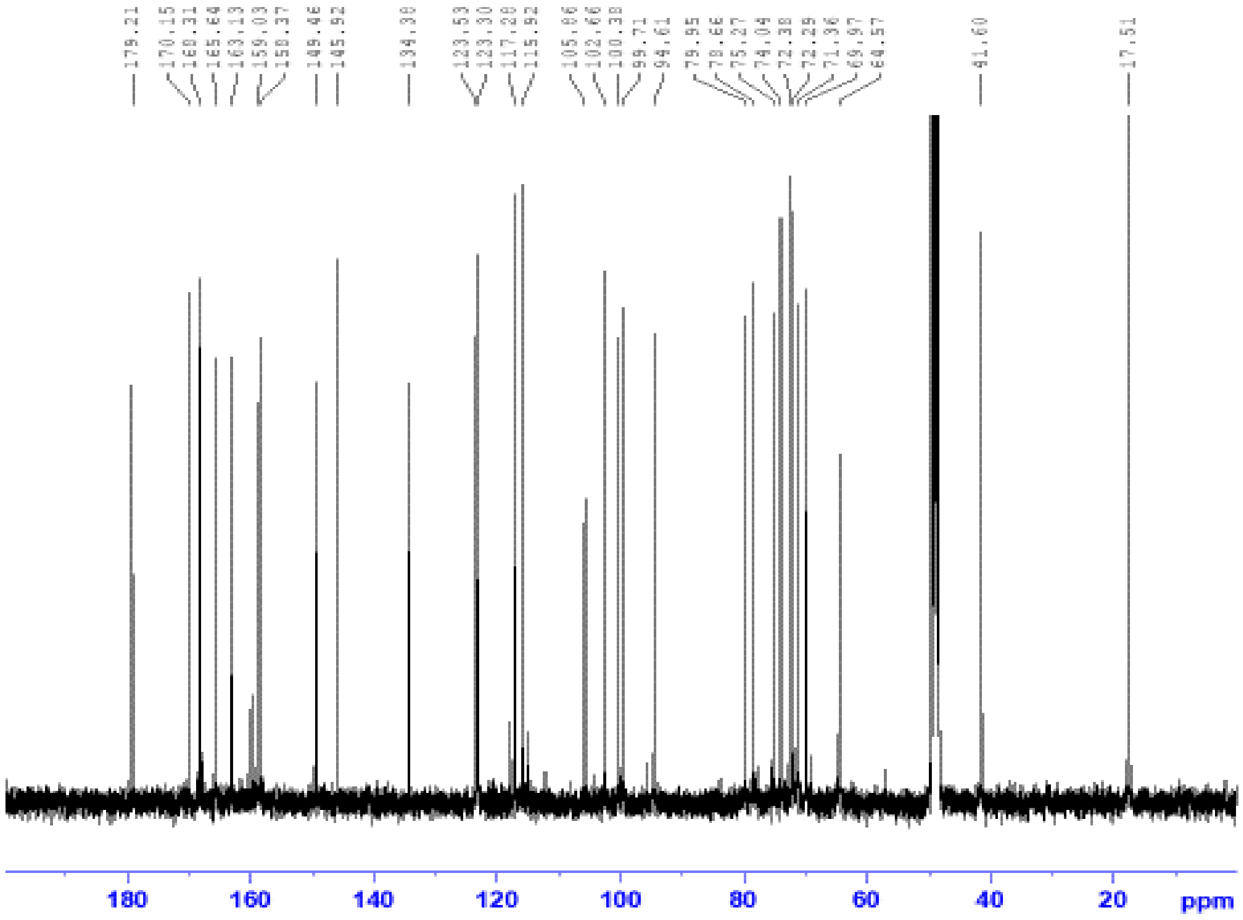

Figure S18. ^{13}C NMR spectrum of **4** in CD_3OD (100 MHz).

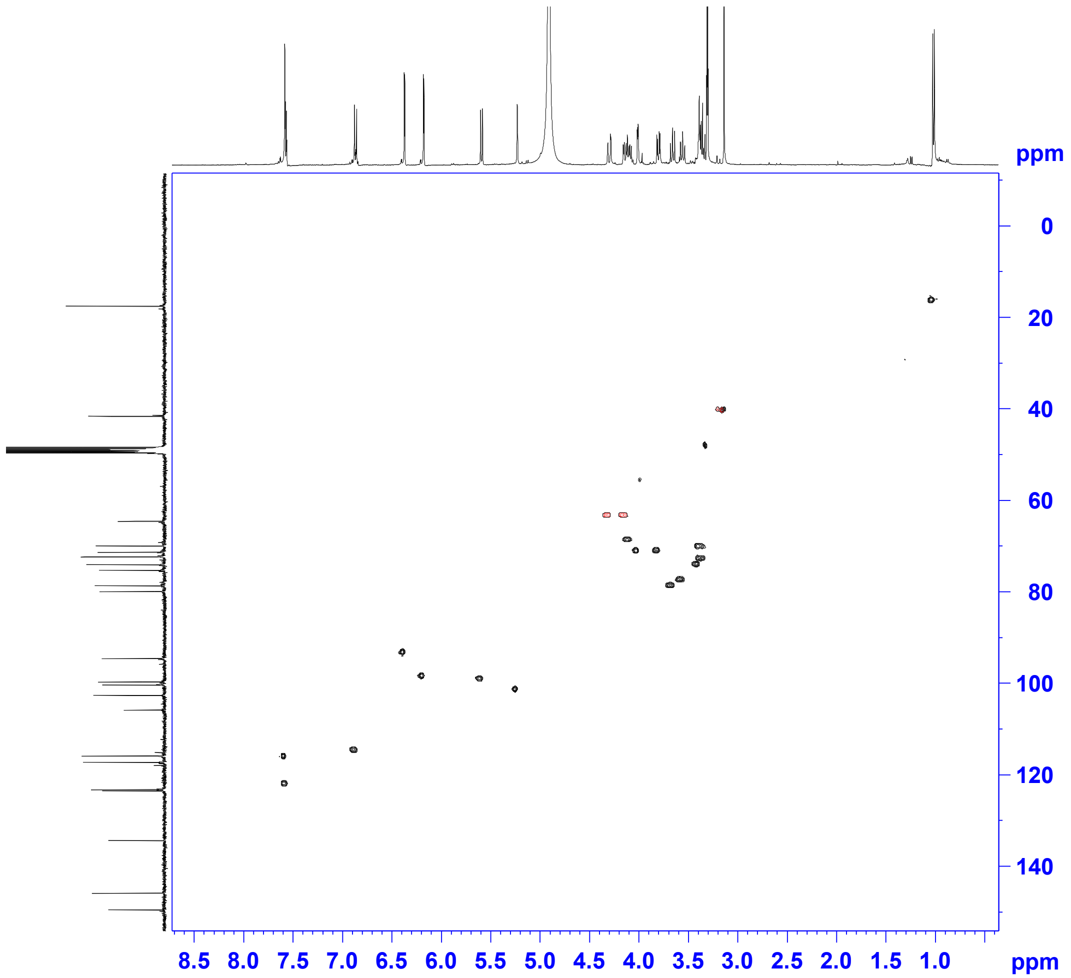

Figure S19. HSQC spectrum of **4** in CD_3OD.

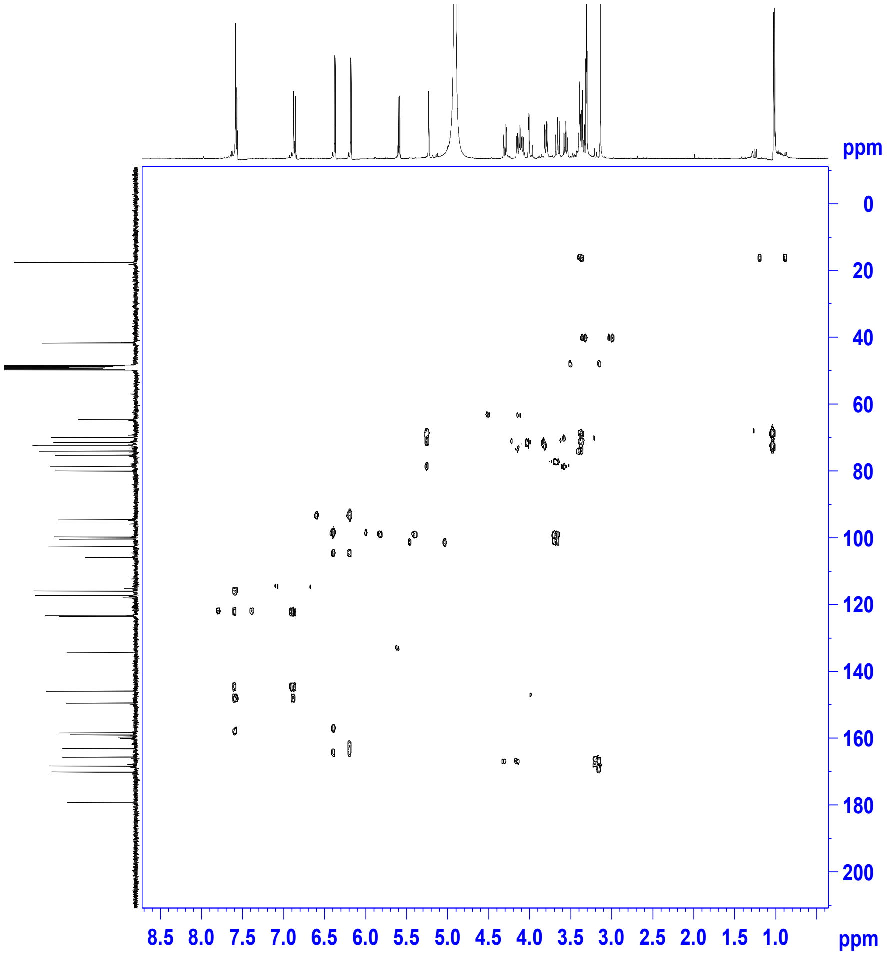

Figure S20. HMBC spectrum of **4** in CD_3OD.

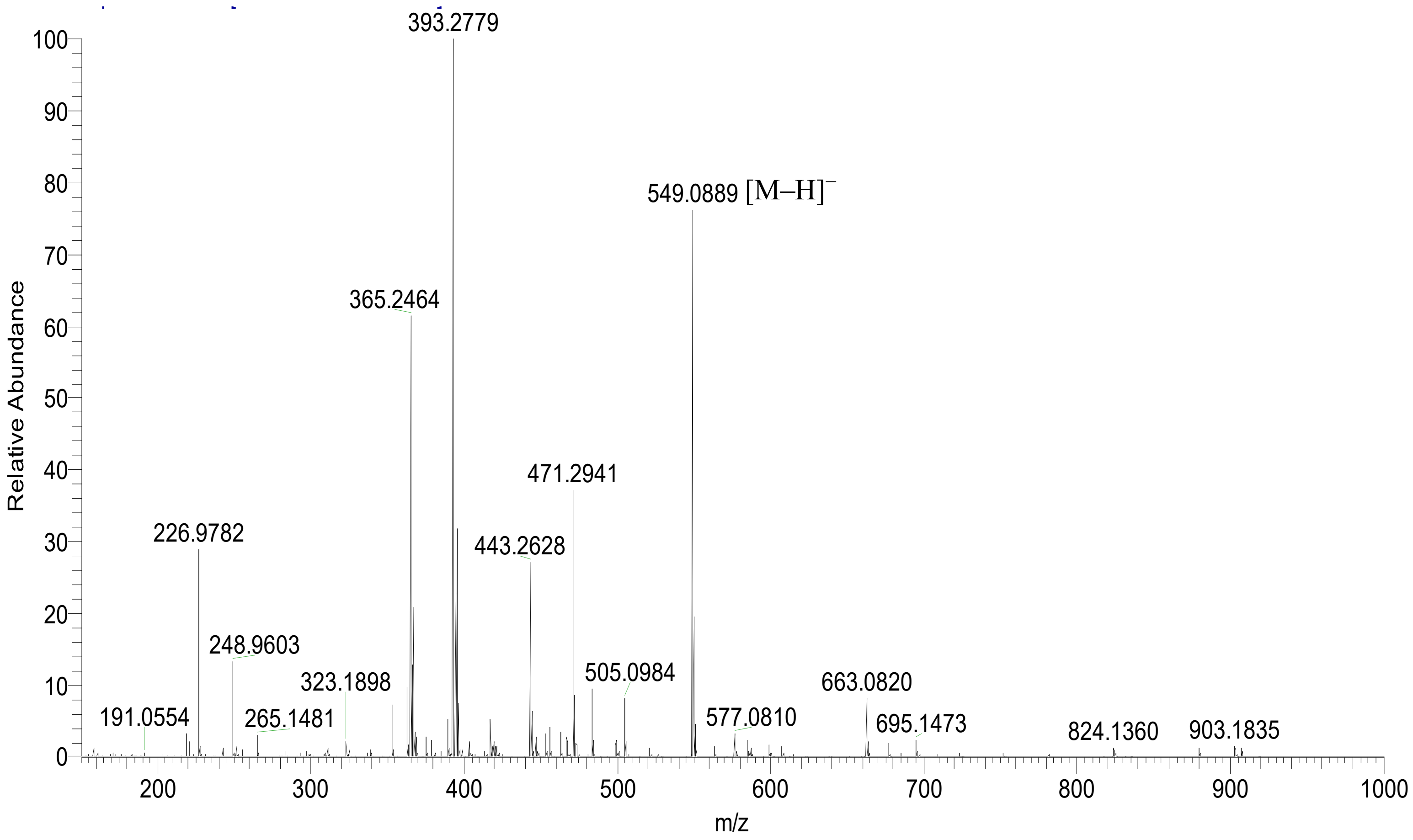

Figure S21. HRESIMS spectrum of **5** (negative mode).

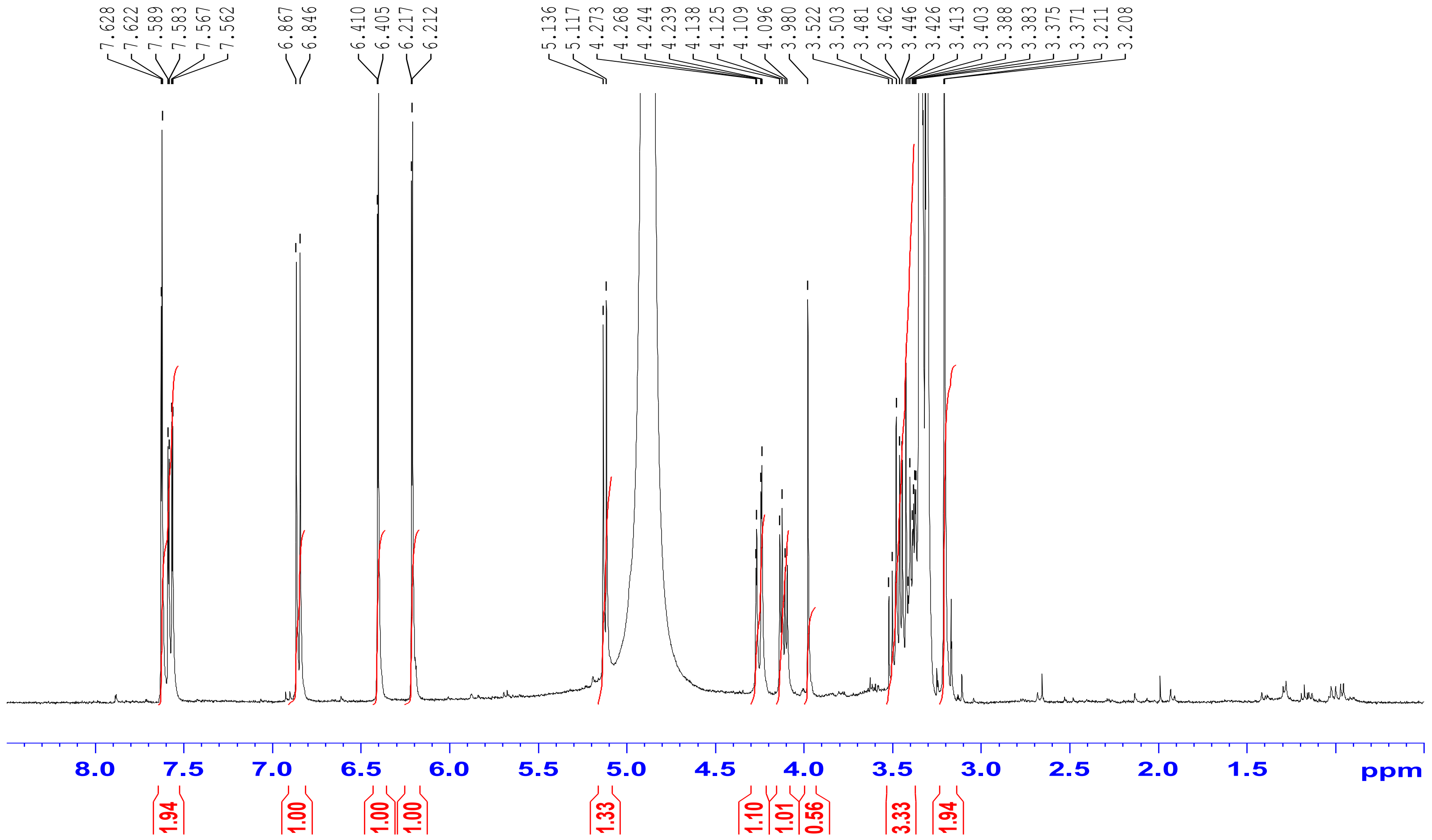

Figure S22. ^{1}H NMR spectrum of **5** in CD_3OD (400 MHz).

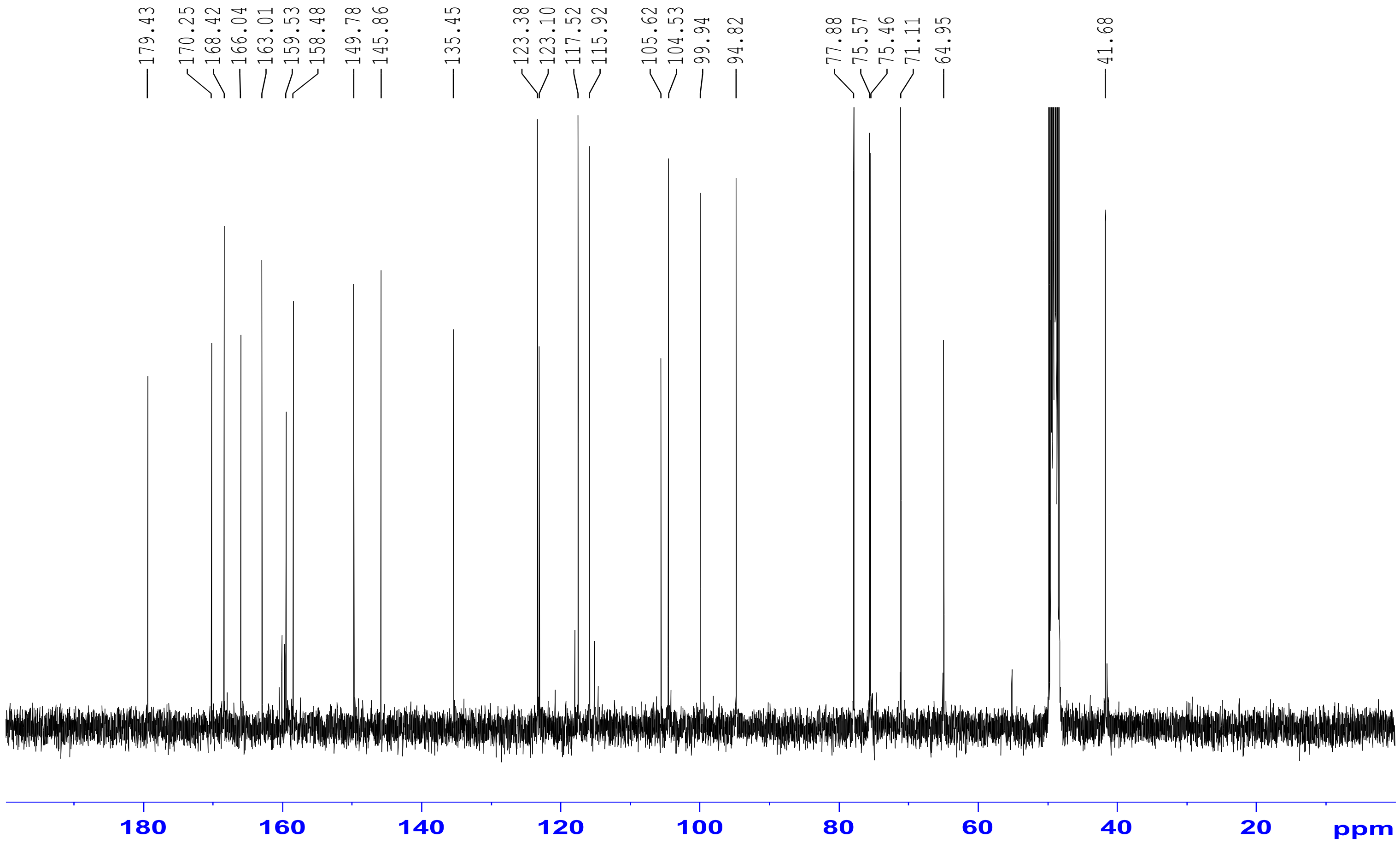

Figure S23. ^{13}C NMR spectrum of **5** in CD_3OD (100 MHz).

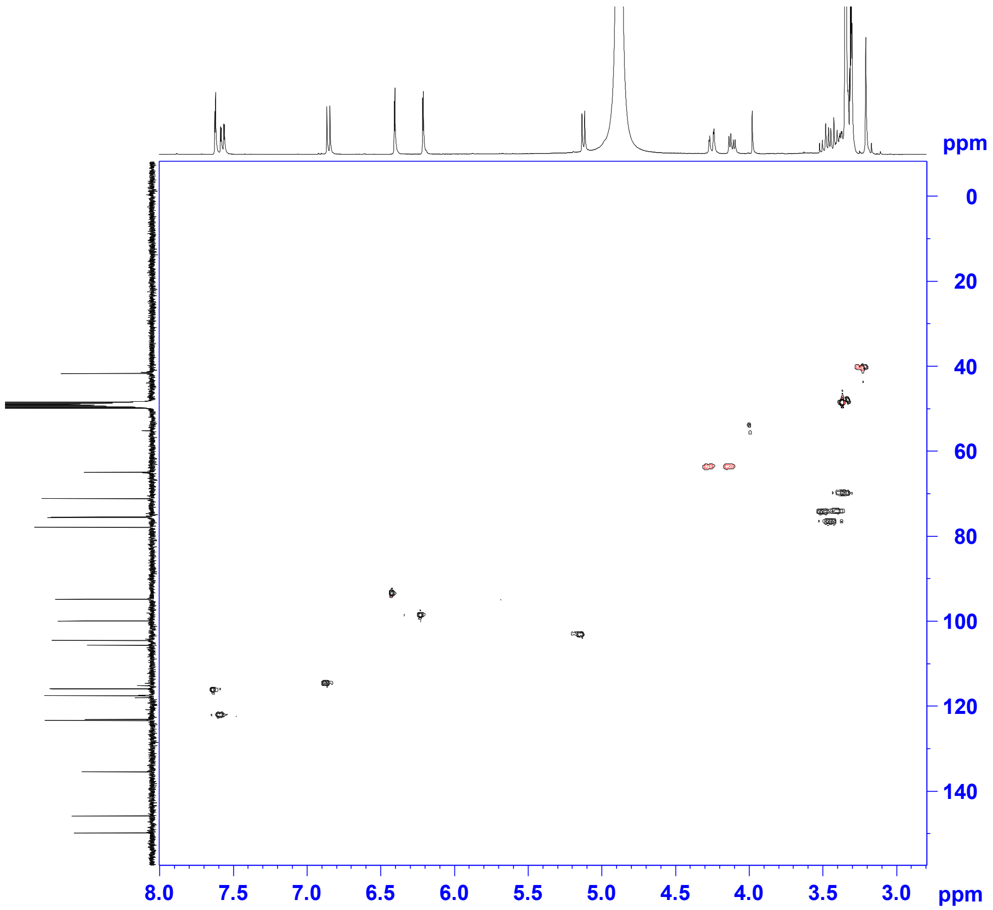

Figure S24. HSQC spectrum of **5** in CD_3OD.

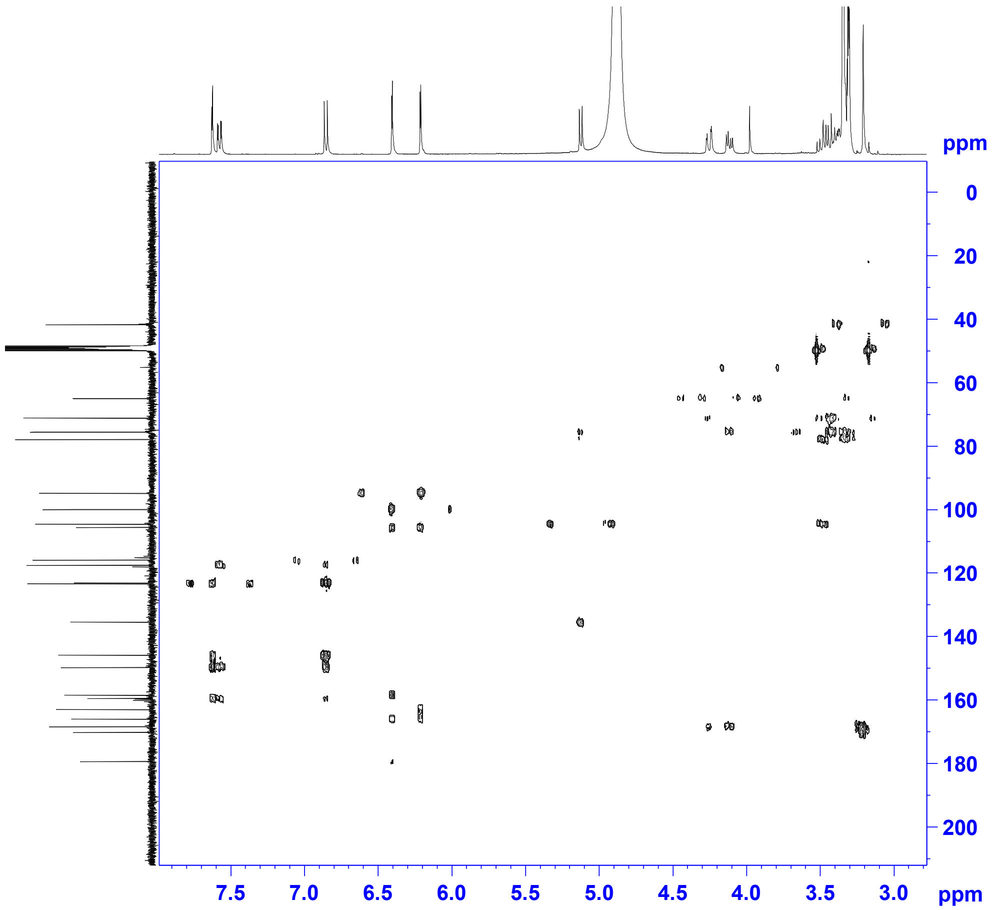

Figure S25. HMBC spectrum of **5** in CD_3OD.

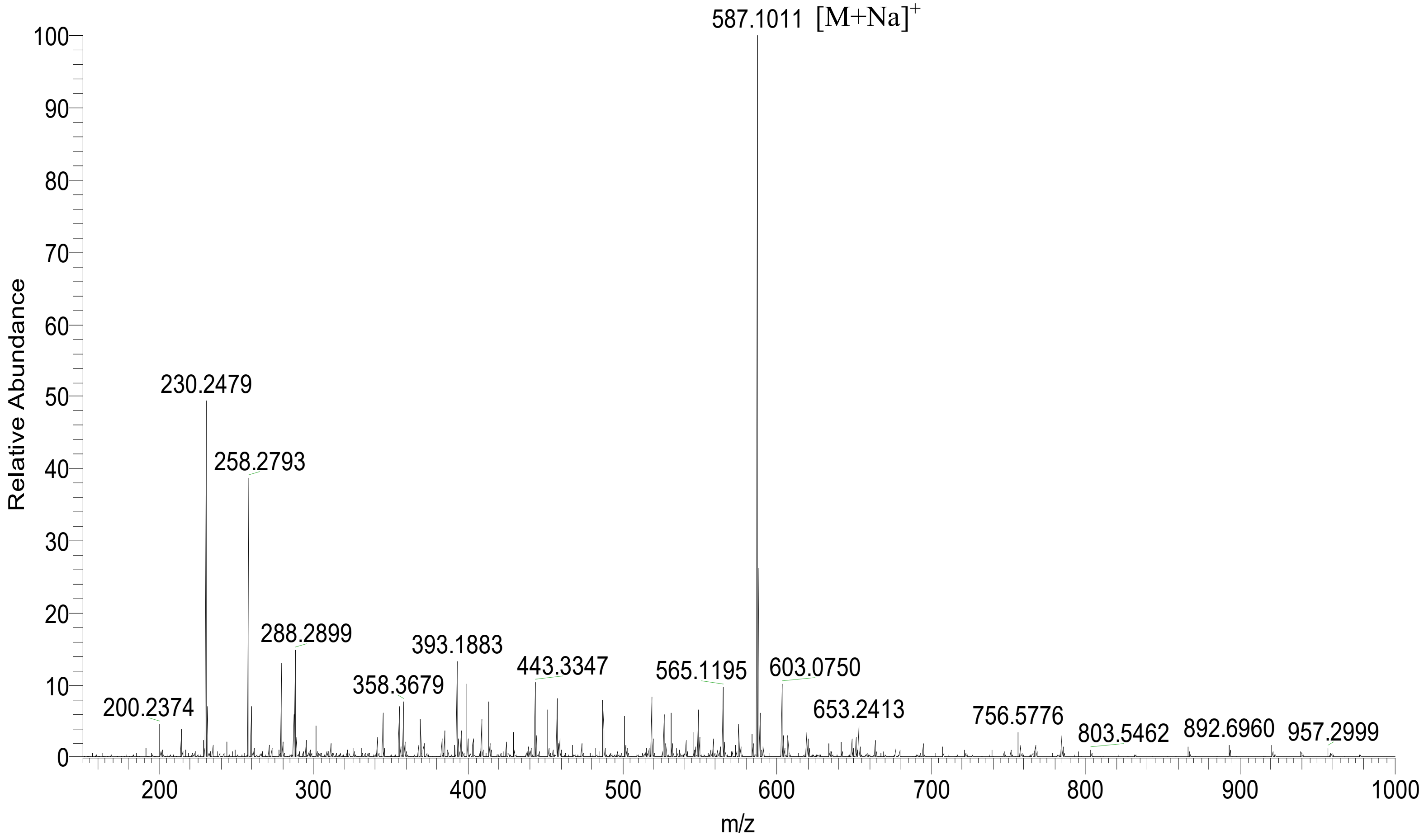

Figure S26. HRESIMS spectrum of **6** (positive mode).

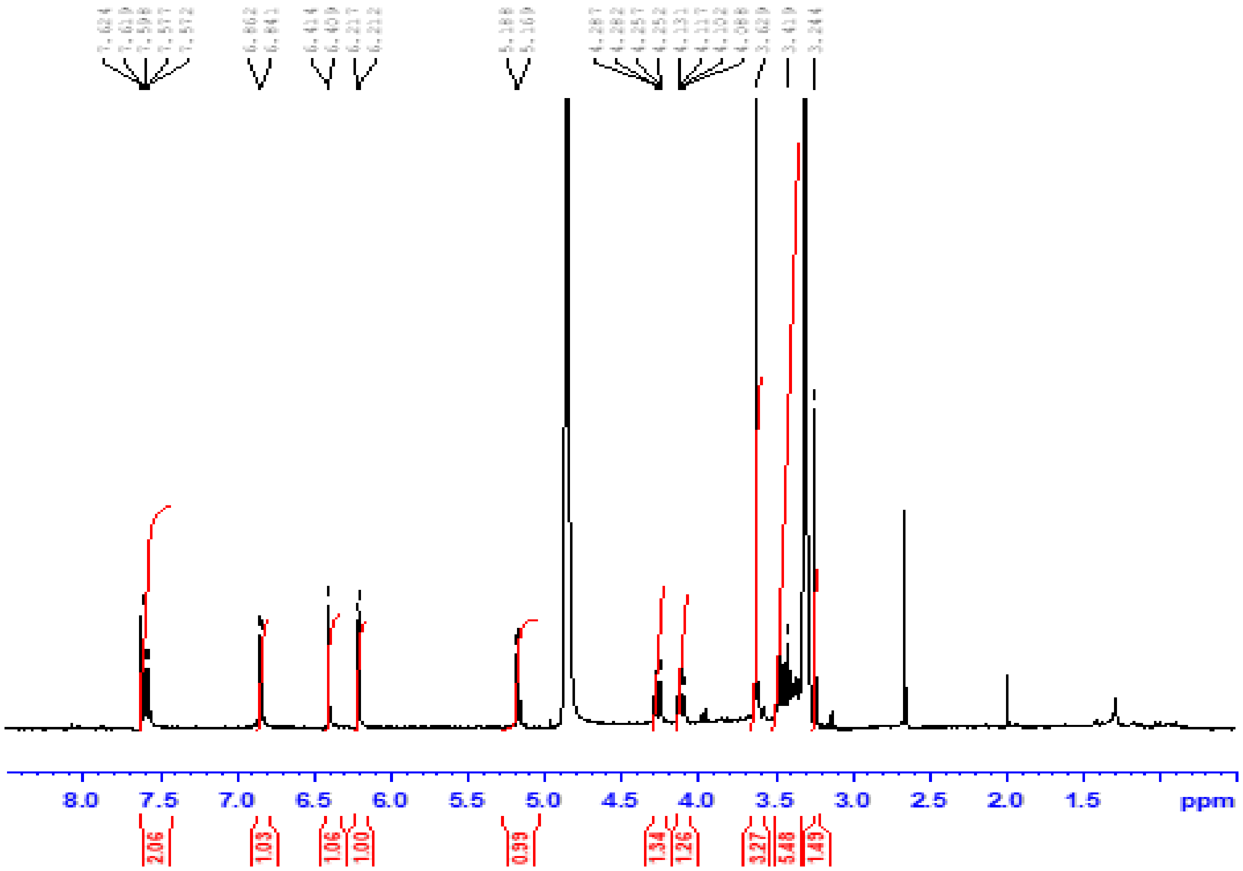

Figure S27. ^{1}H NMR spectrum of **6** in CD_3OD (400 MHz).

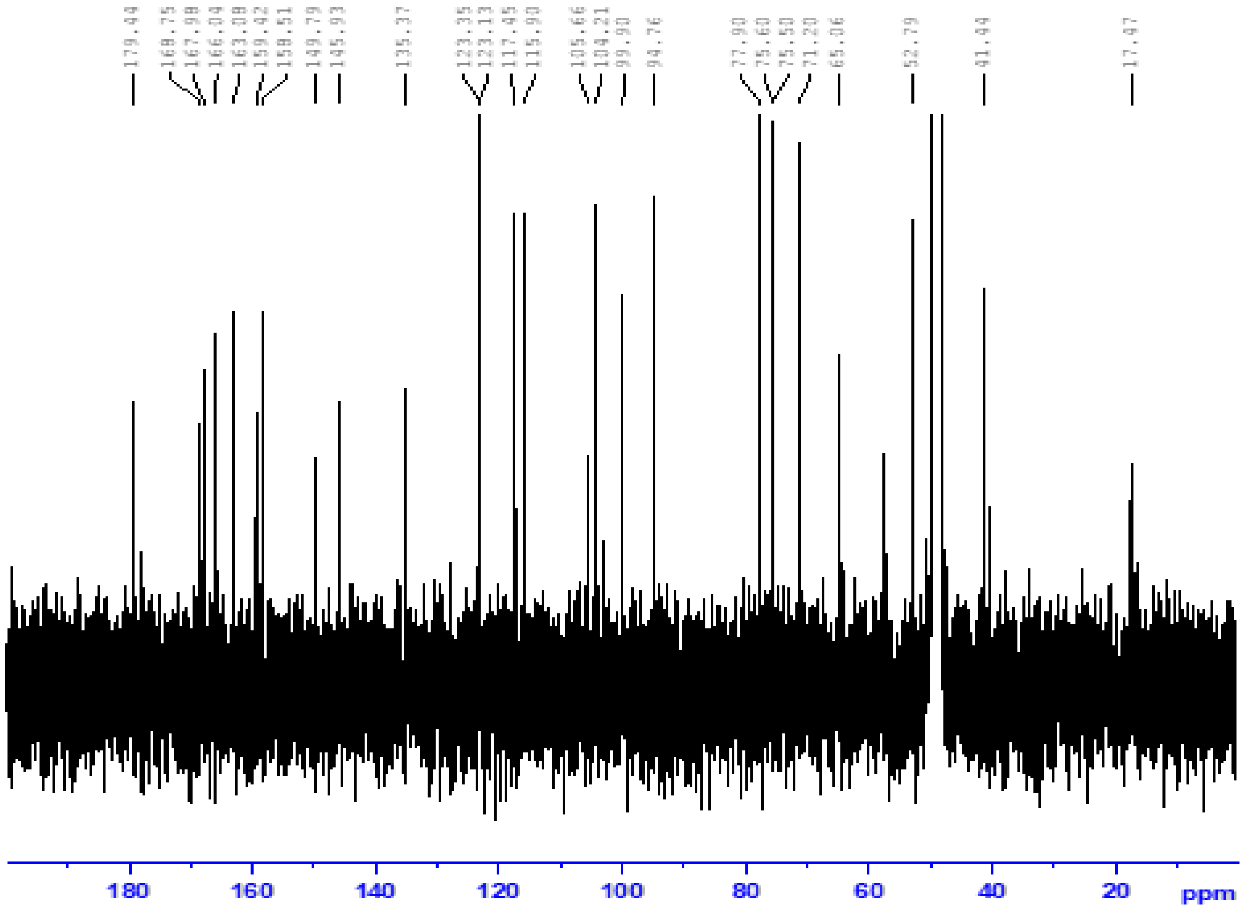

Figure S28. ^{13}C NMR spectrum of **6** in CD_3OD (100 MHz).

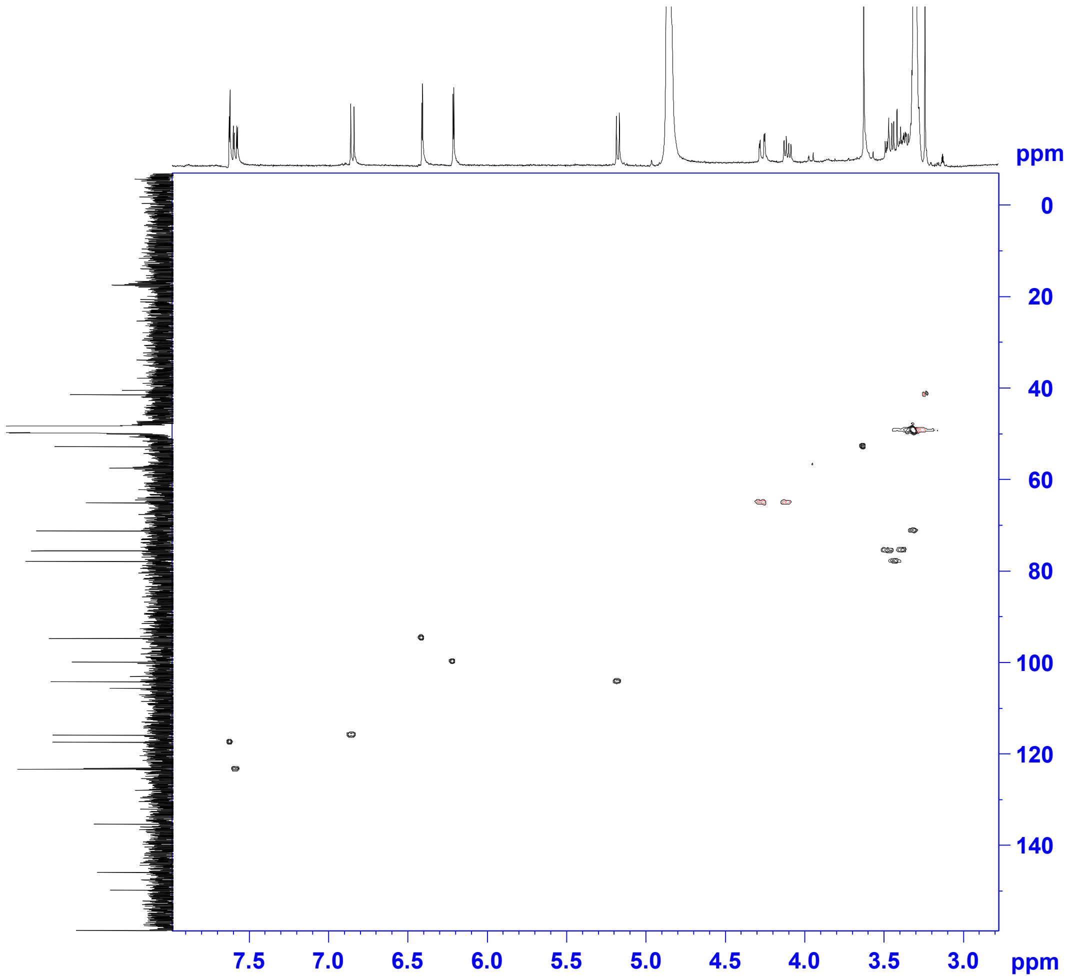

Figure S29. HSQC spectrum of **6** in CD_3OD.

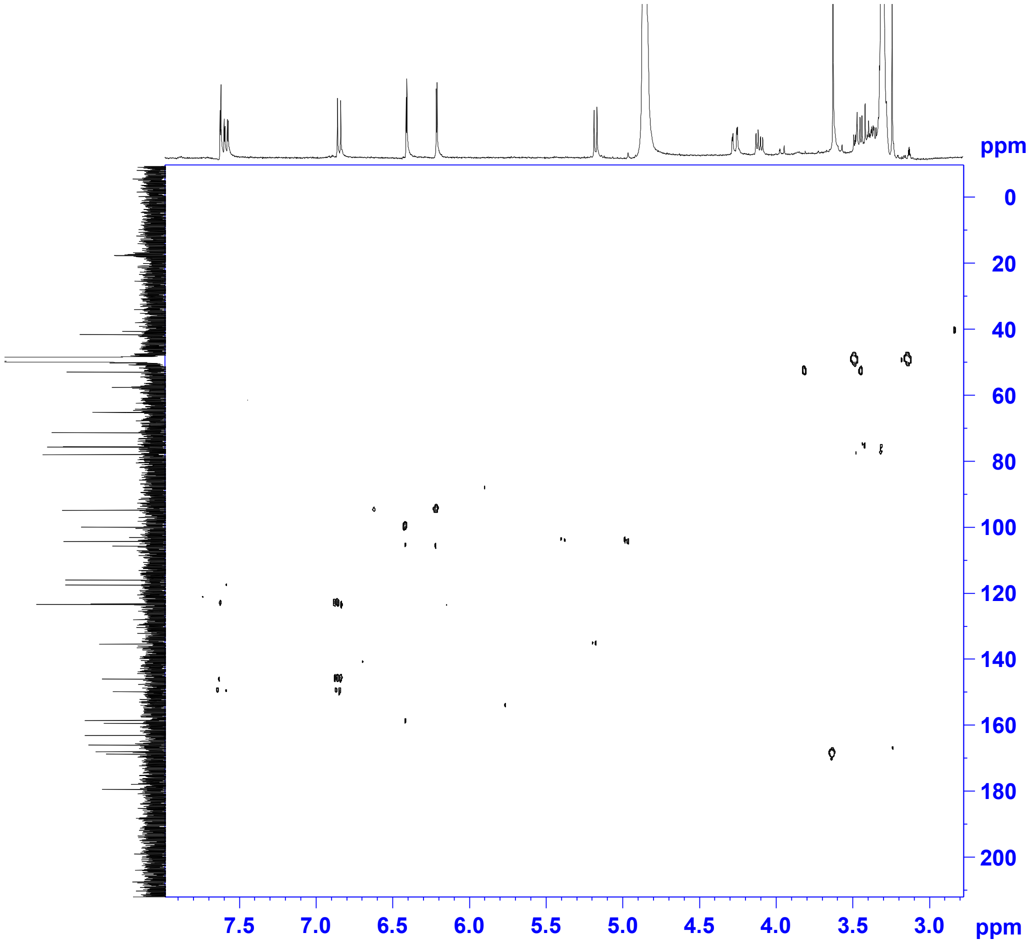

Figure S30. HMBC spectrum of **6** in CD_3OD.

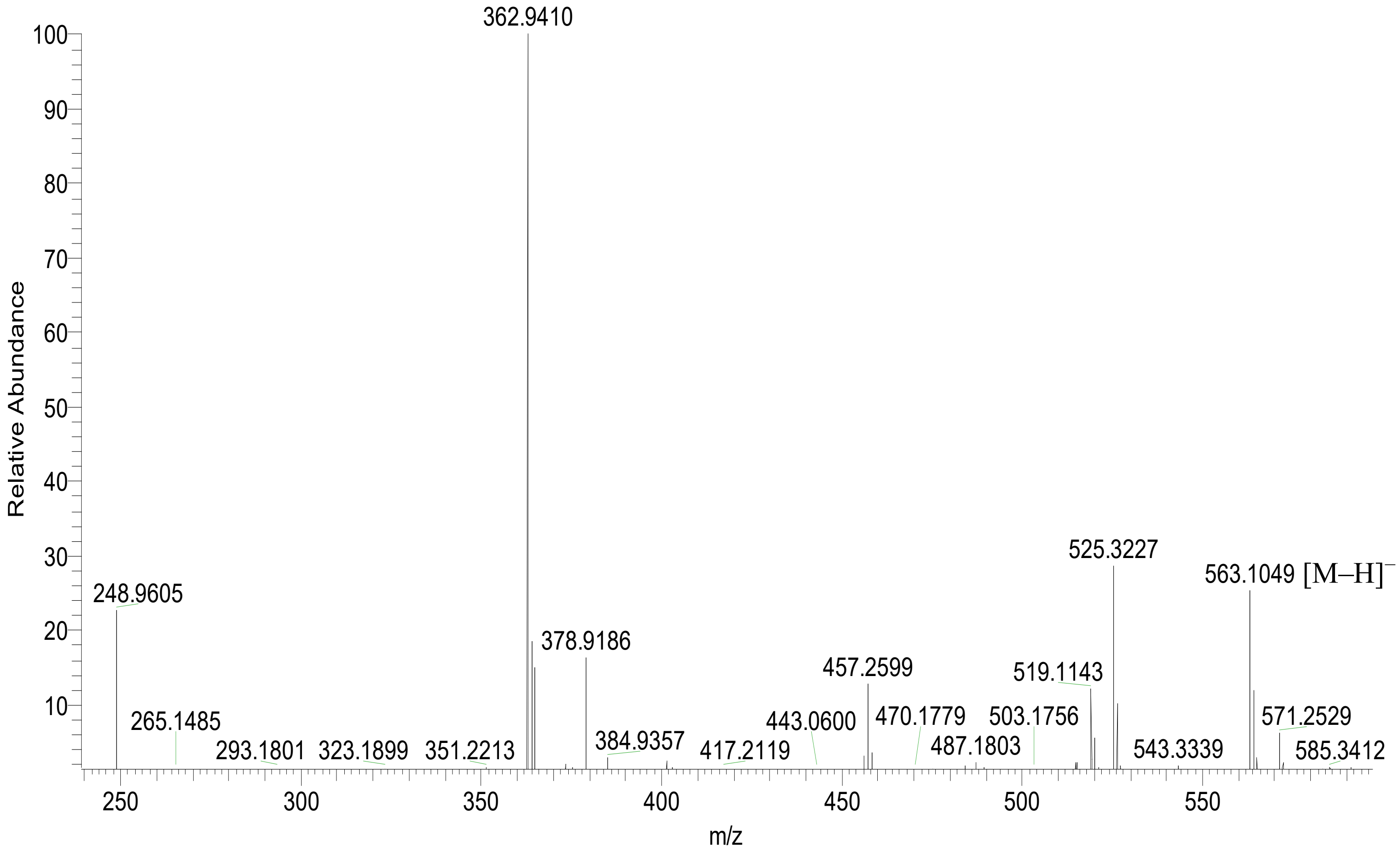

Figure S31. HRESIMS spectrum of **7** (negative mode).

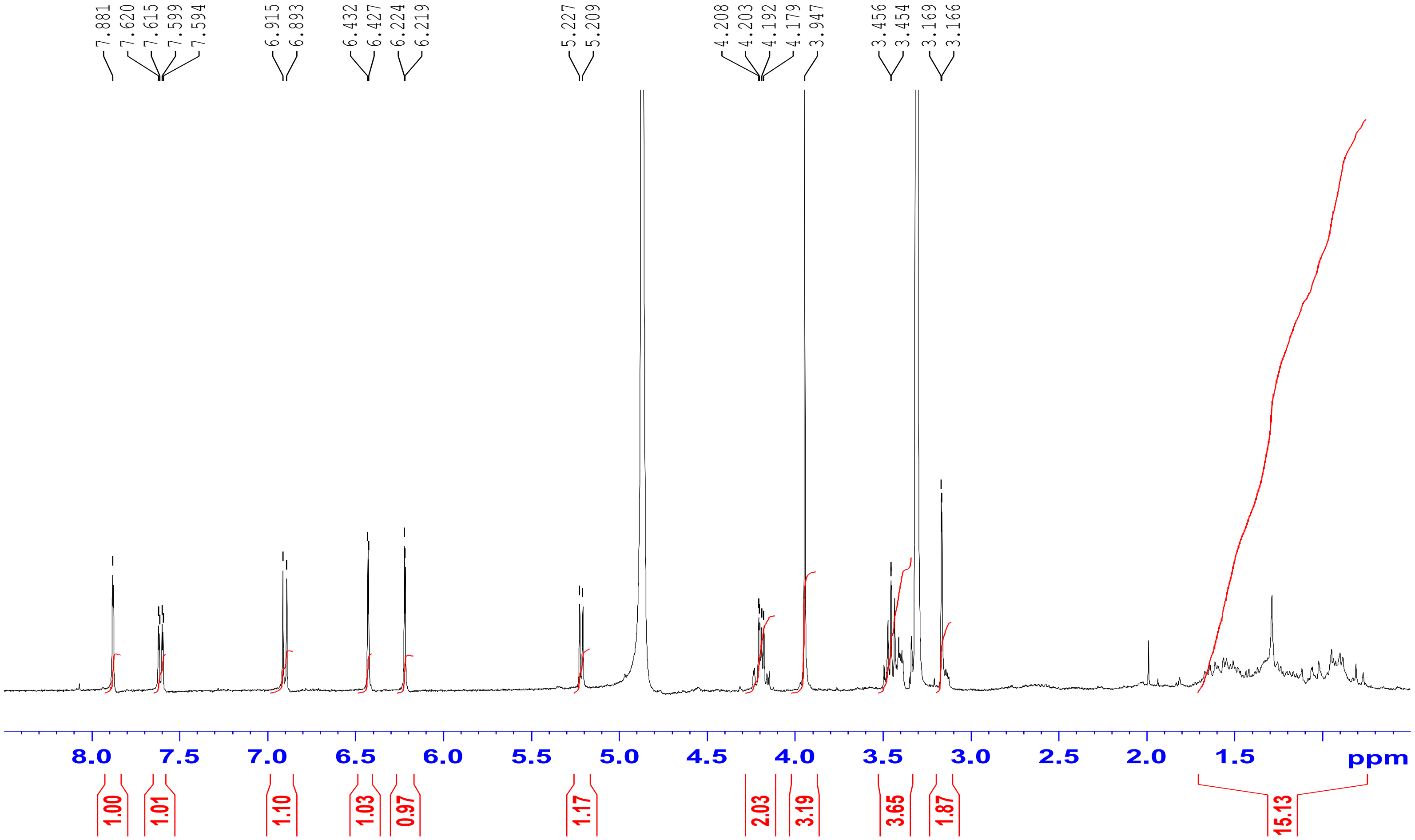

Figure S32. ^{1}H NMR spectrum of **7** in CD_3OD (400 MHz).

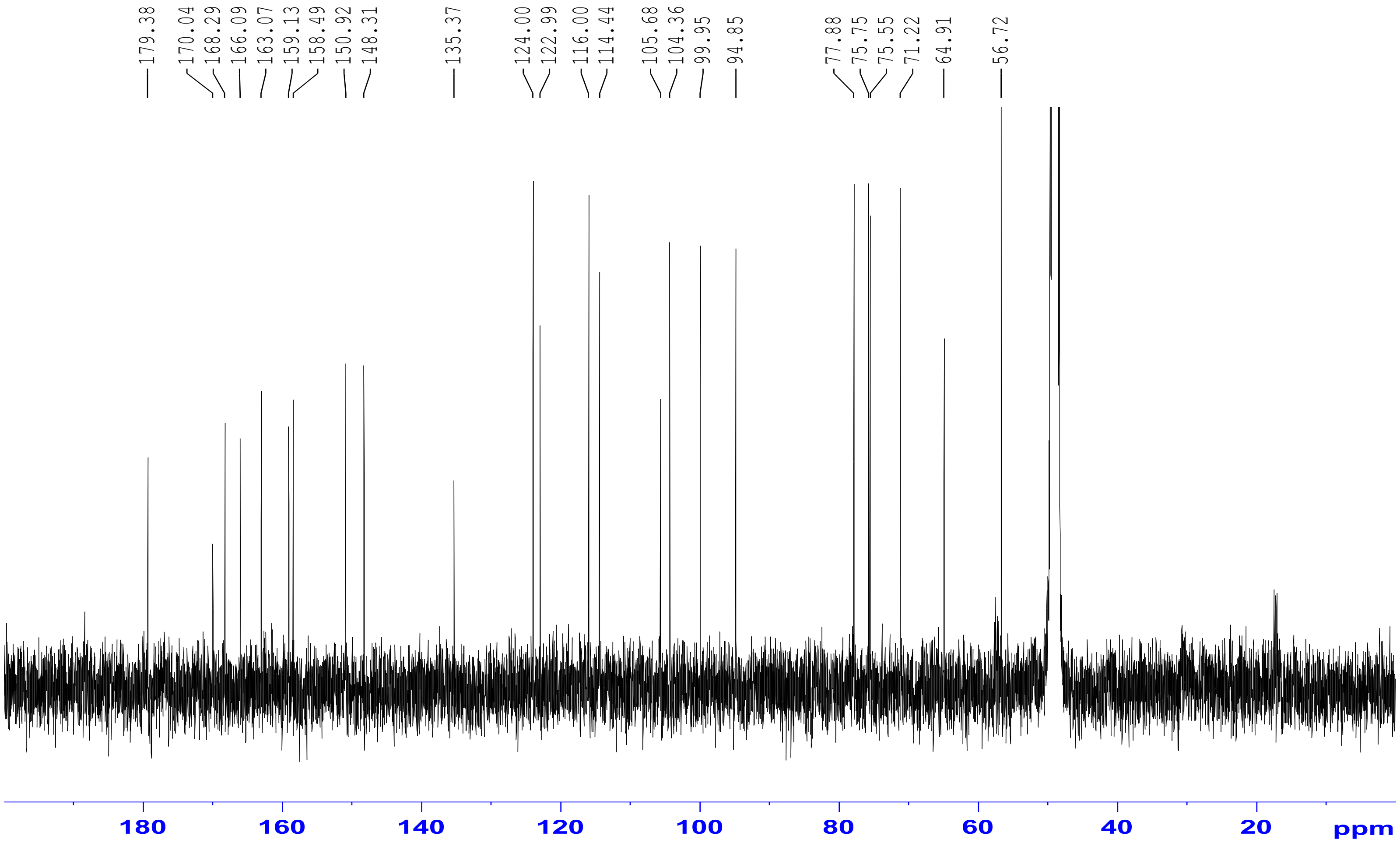

Figure S33. ^{13}C NMR spectrum of **7** in CD_3OD (100 MHz).

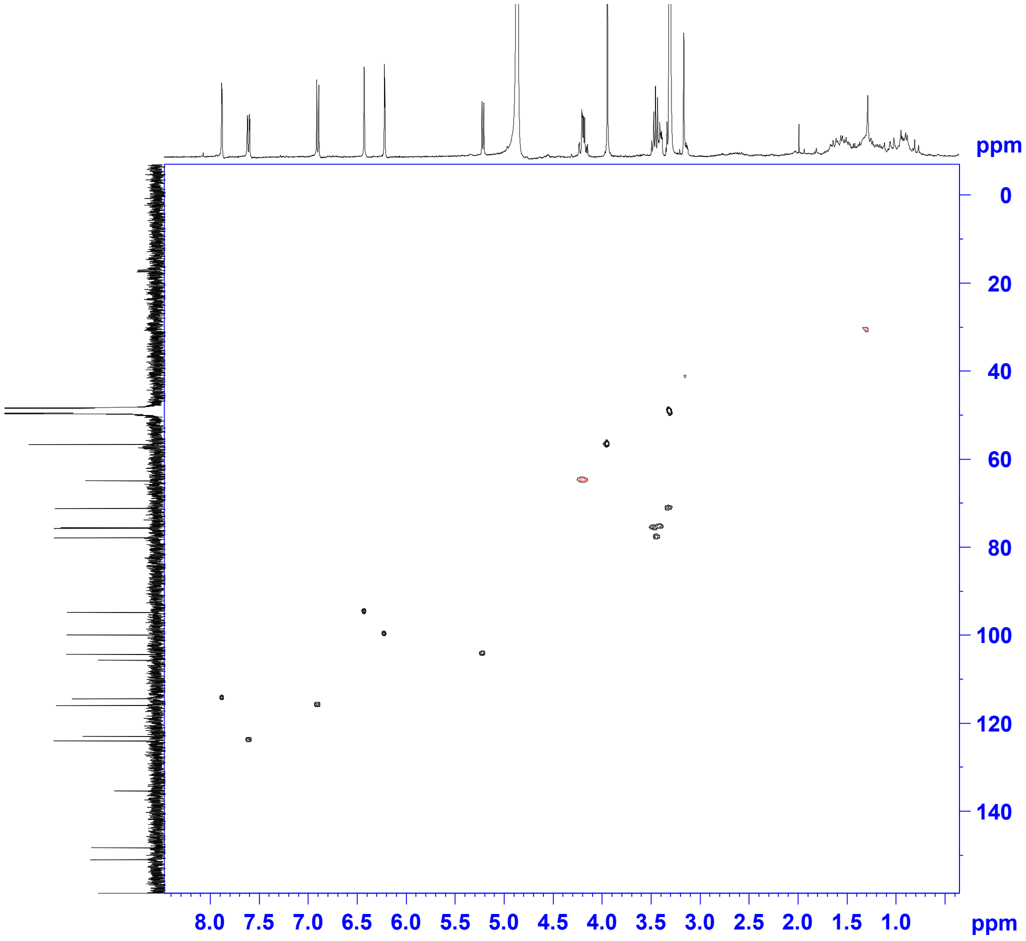

Figure S34. HSQC spectrum of **7** in CD_3OD.

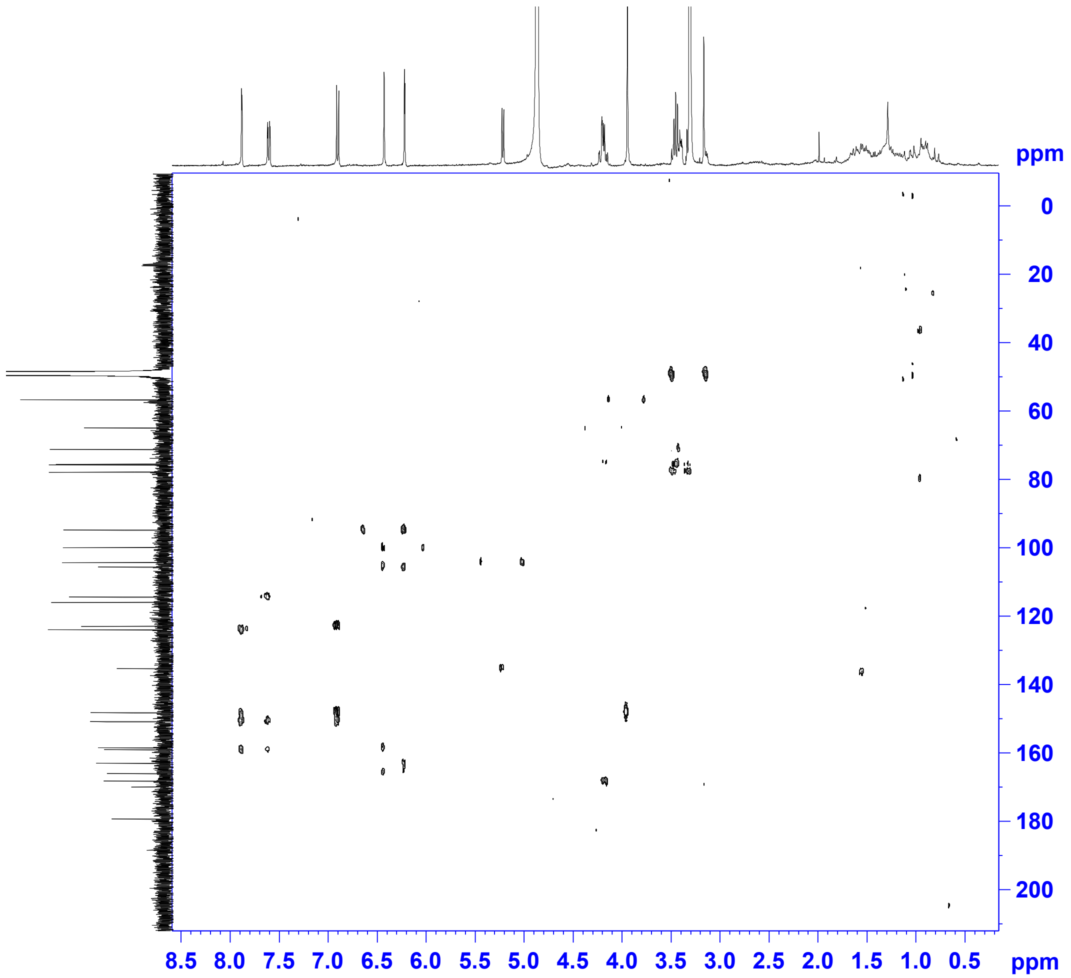

Figure S35. HMBC spectrum of **7** in CD_3OD.

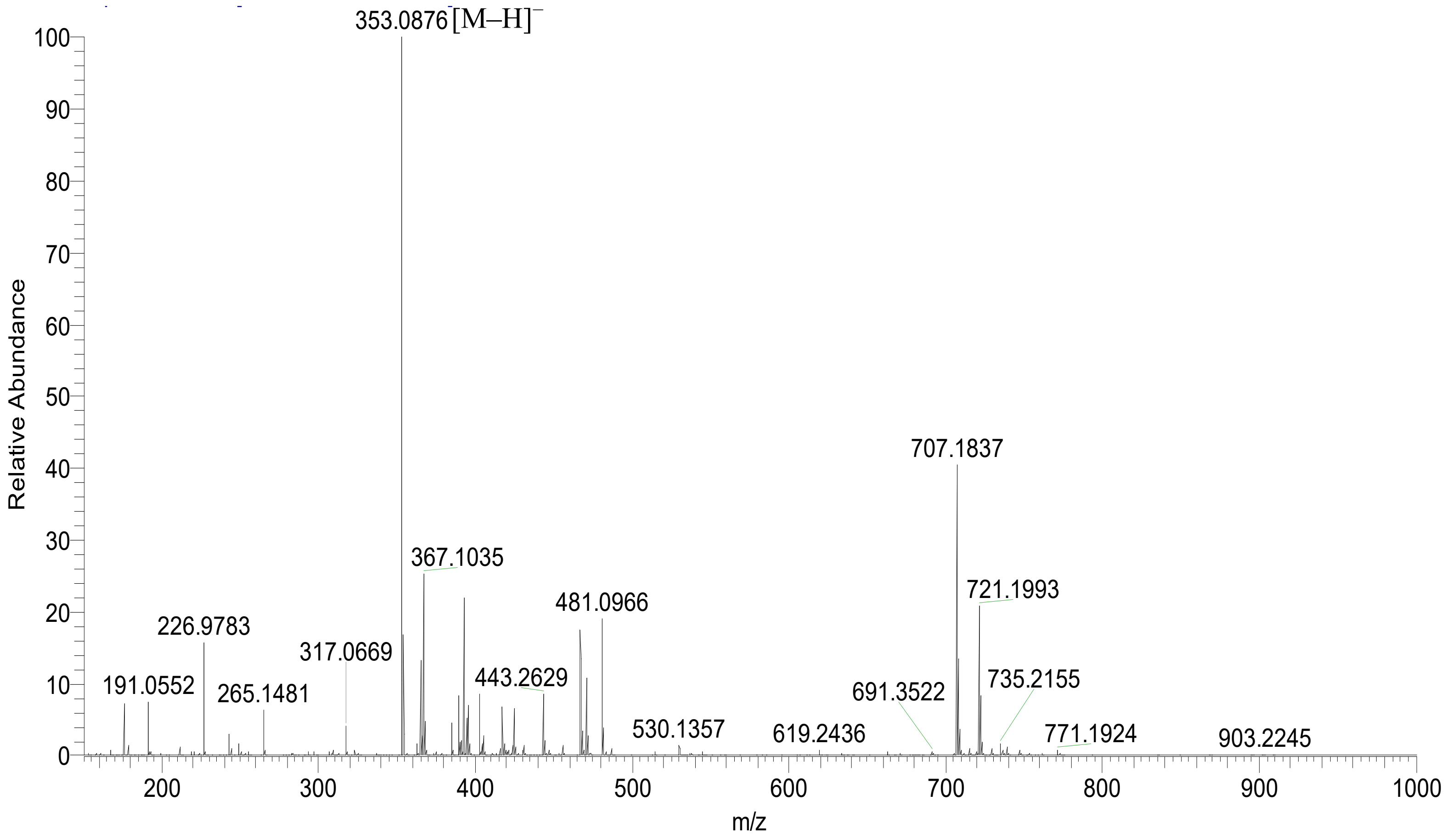

Figure S36. HRESIMS spectrum of **8** (negative mode).

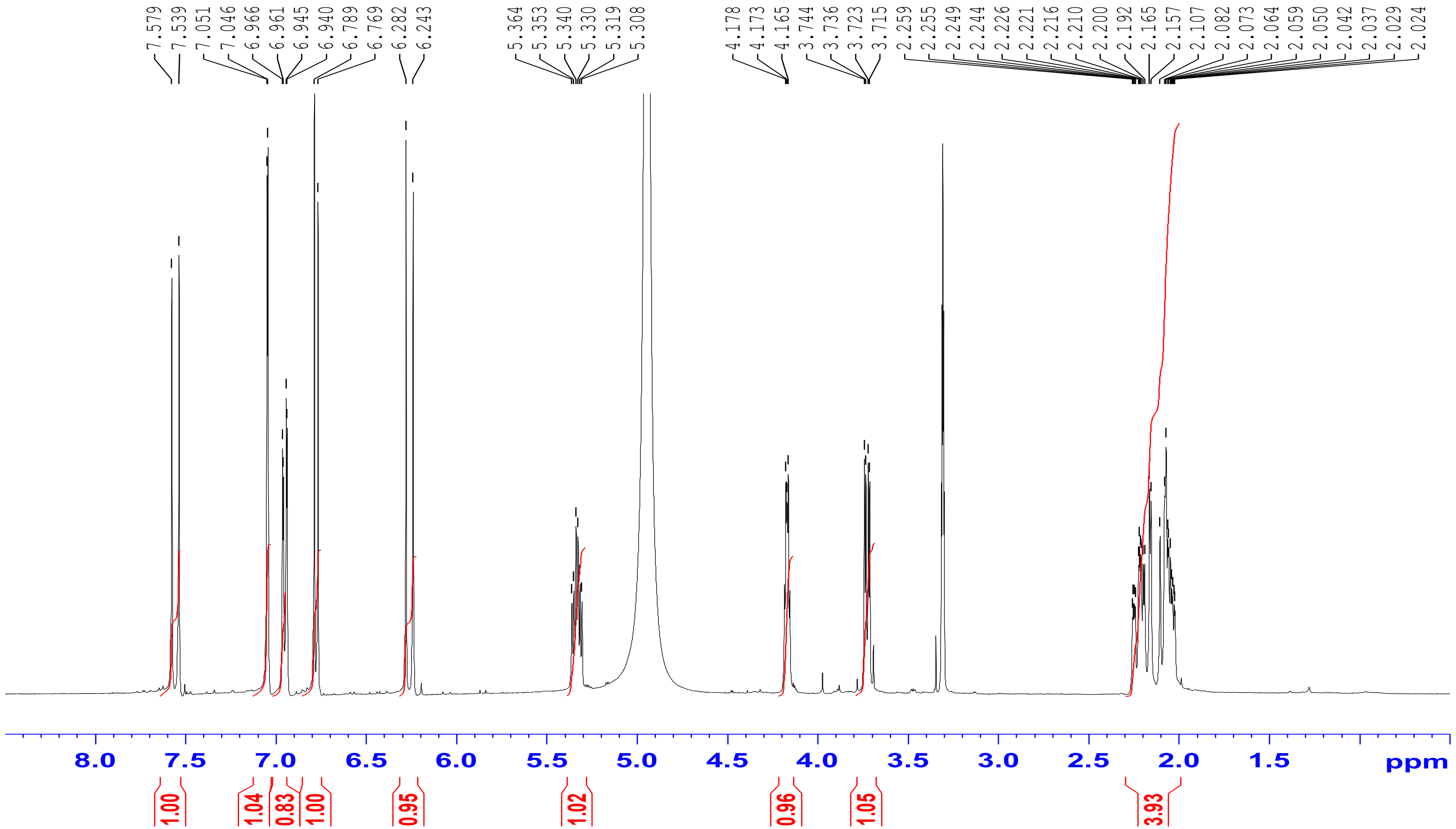

Figure S37. ^{1}H NMR spectrum of **8** in CD_3OD (400 MHz).

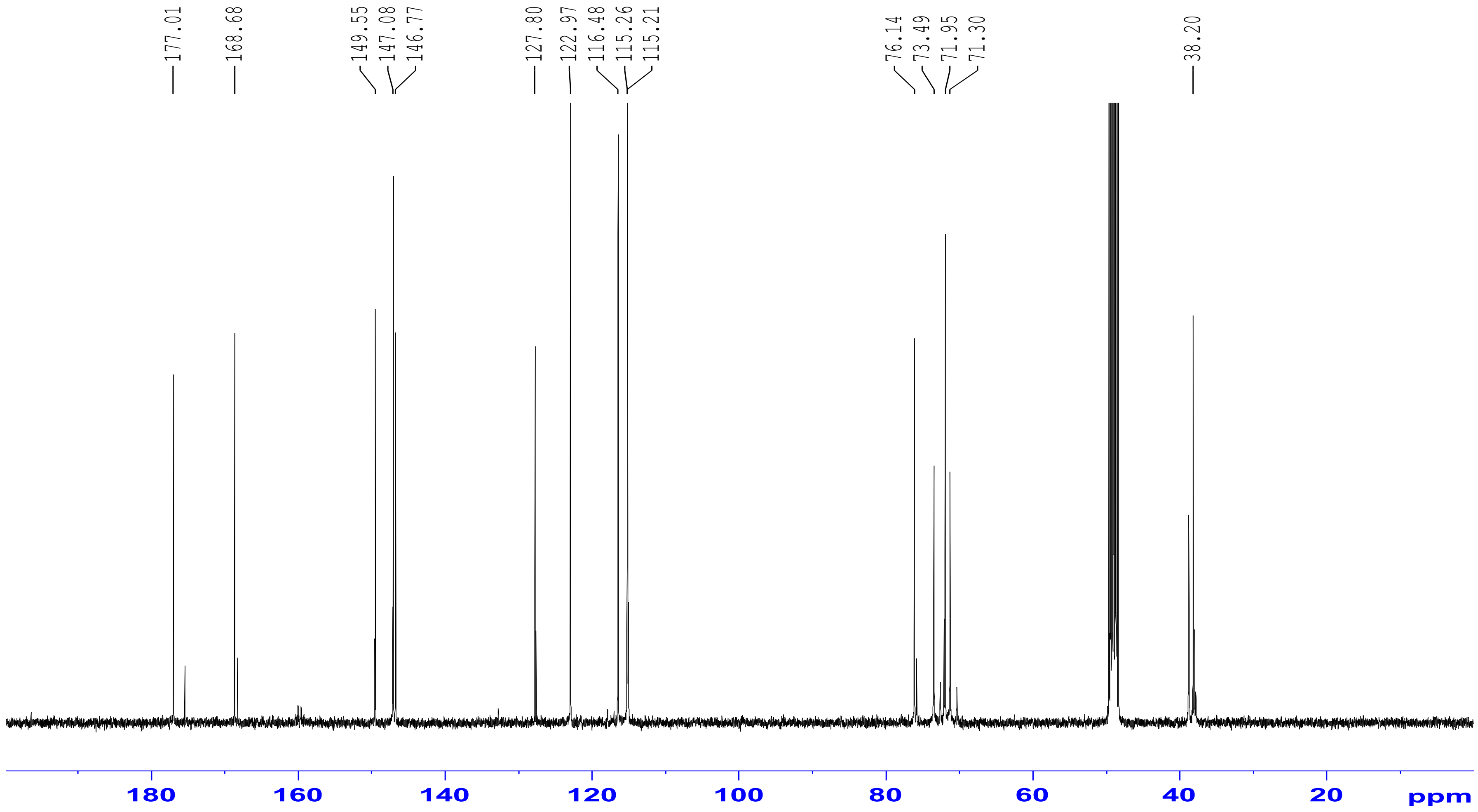

Figure S38. ^{13}C NMR spectrum of **8** in CD_3OD (100 MHz).

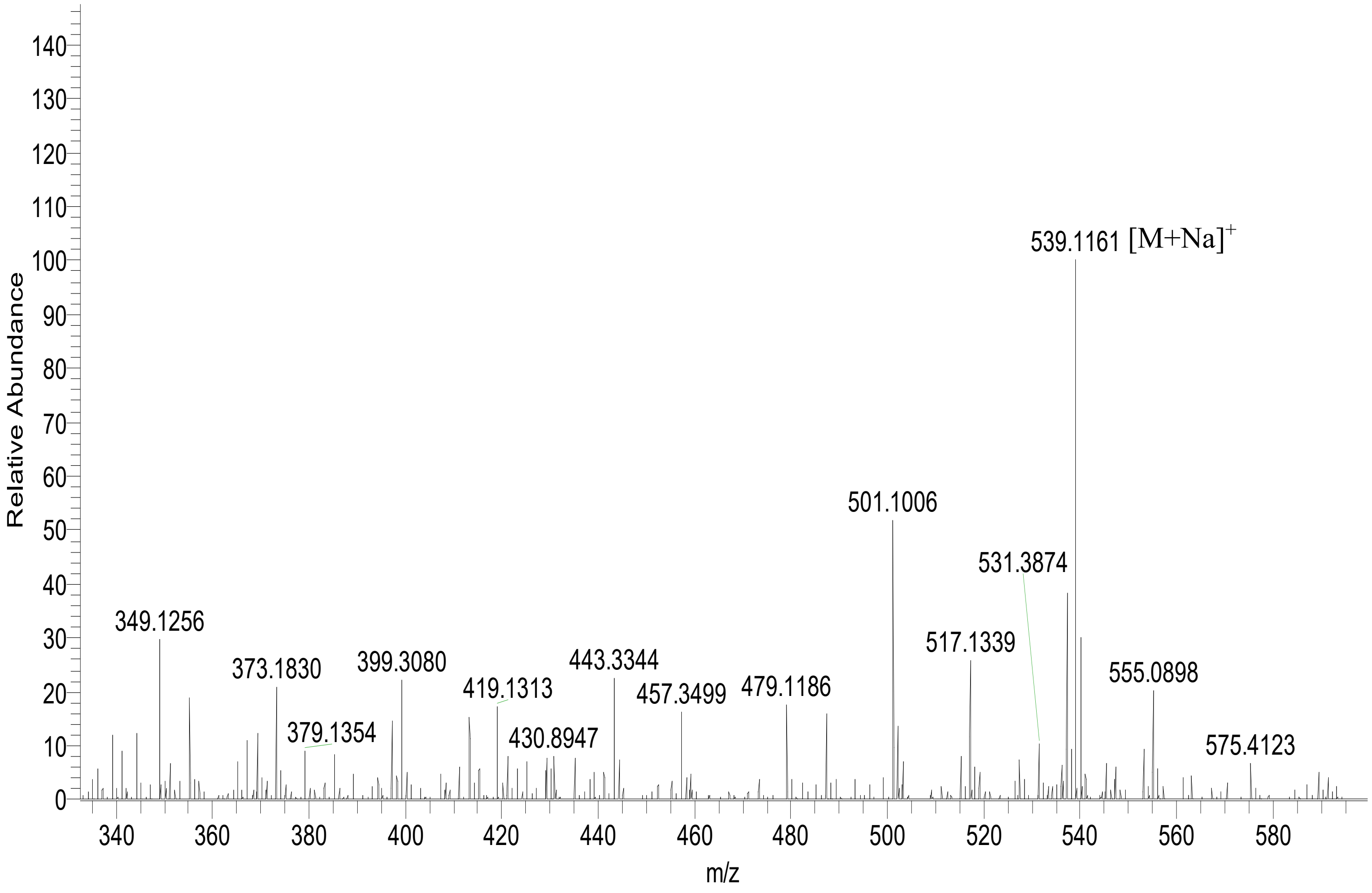

Figure S39. HRESIMS spectrum of **9** (positive mode).

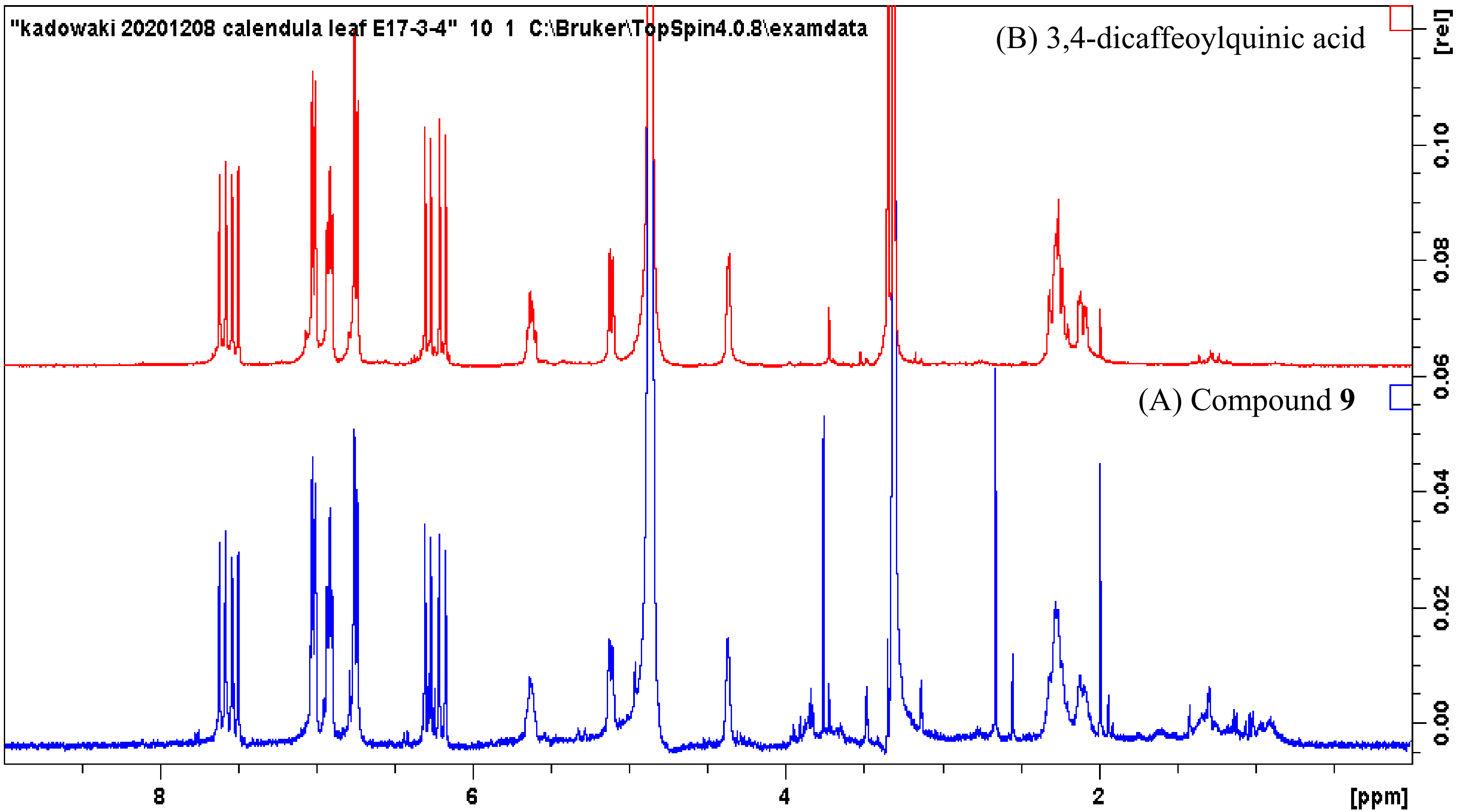

Figure S40. ^{1}H NMR spectra of **9** (A) and 3,4-dicaffeoylquinic acid (B) in CD_3OD (400 MHz).

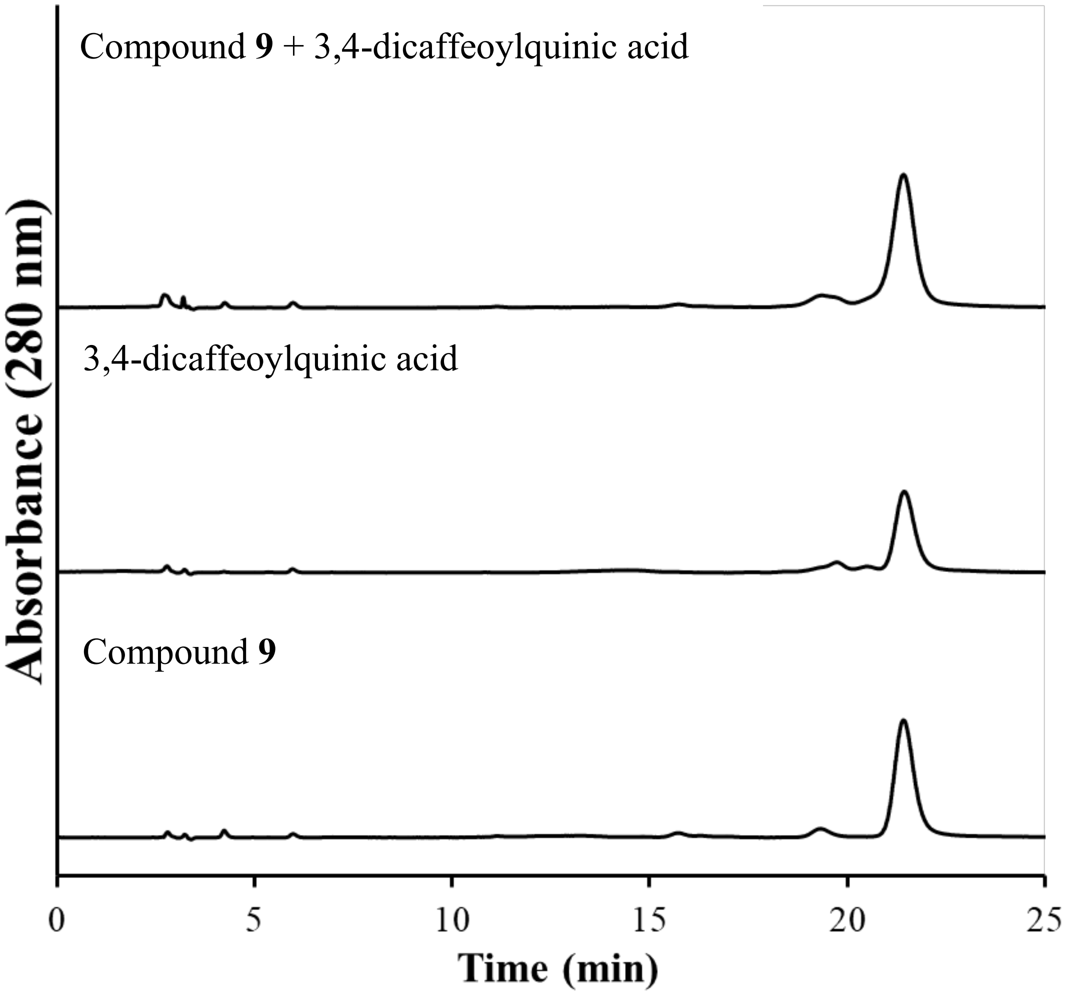

Figure S41. HPLC chromatograms of **9** and 3,4-dicaffeoylquinic acid.

<HPLC analysis condition>

Column	:	Osaka Soda CAPCELL PAK C18 UG120 (5 µm, φ4.6 × 250 mm)
Solvent	:	H_2O:MeCN = 80:20 (0.1% TFA)
Flow rate	:	1.0 mL/min
Detection	:	280 nm

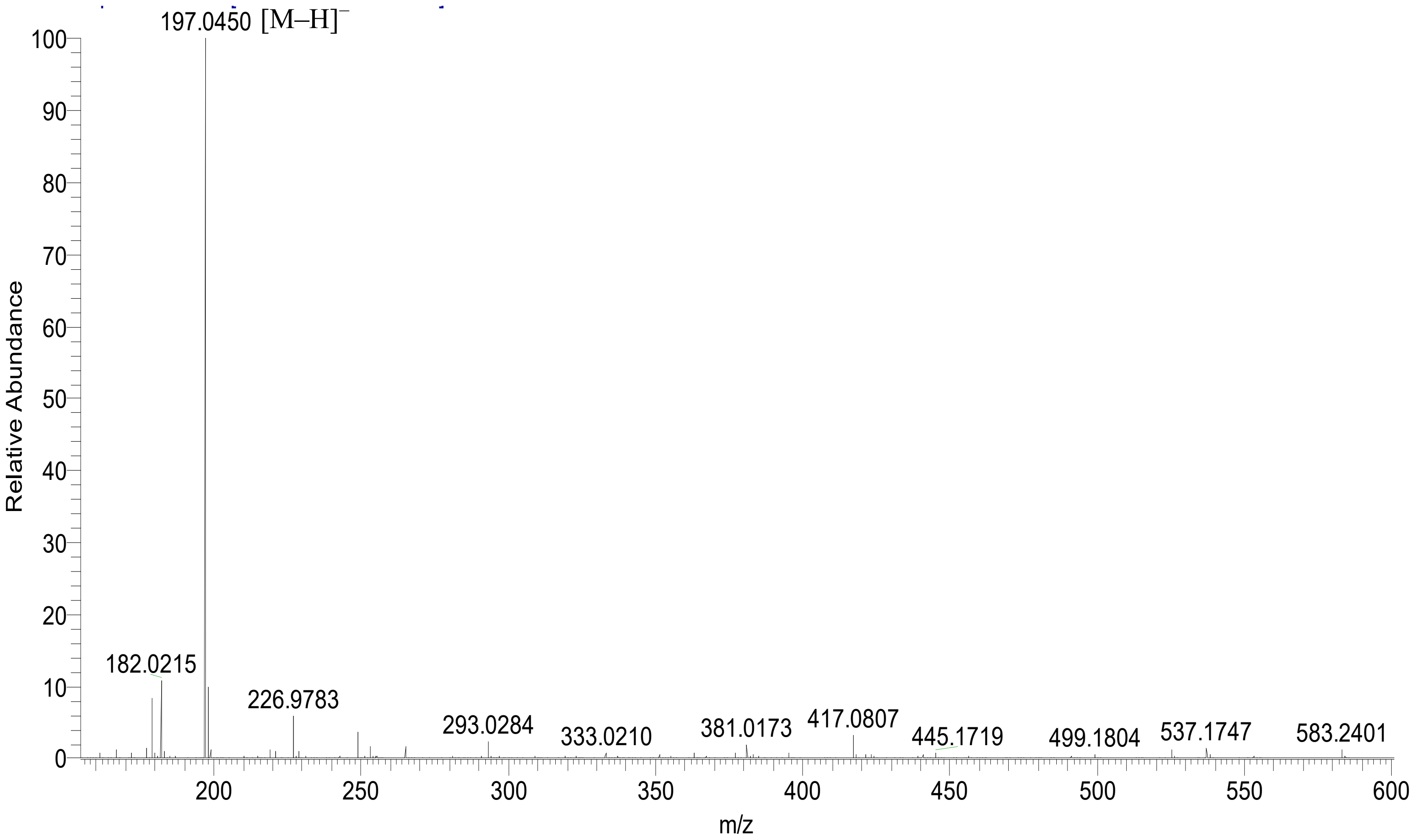

Figure S42. HRESIMS spectrum of **10** (negative mode).

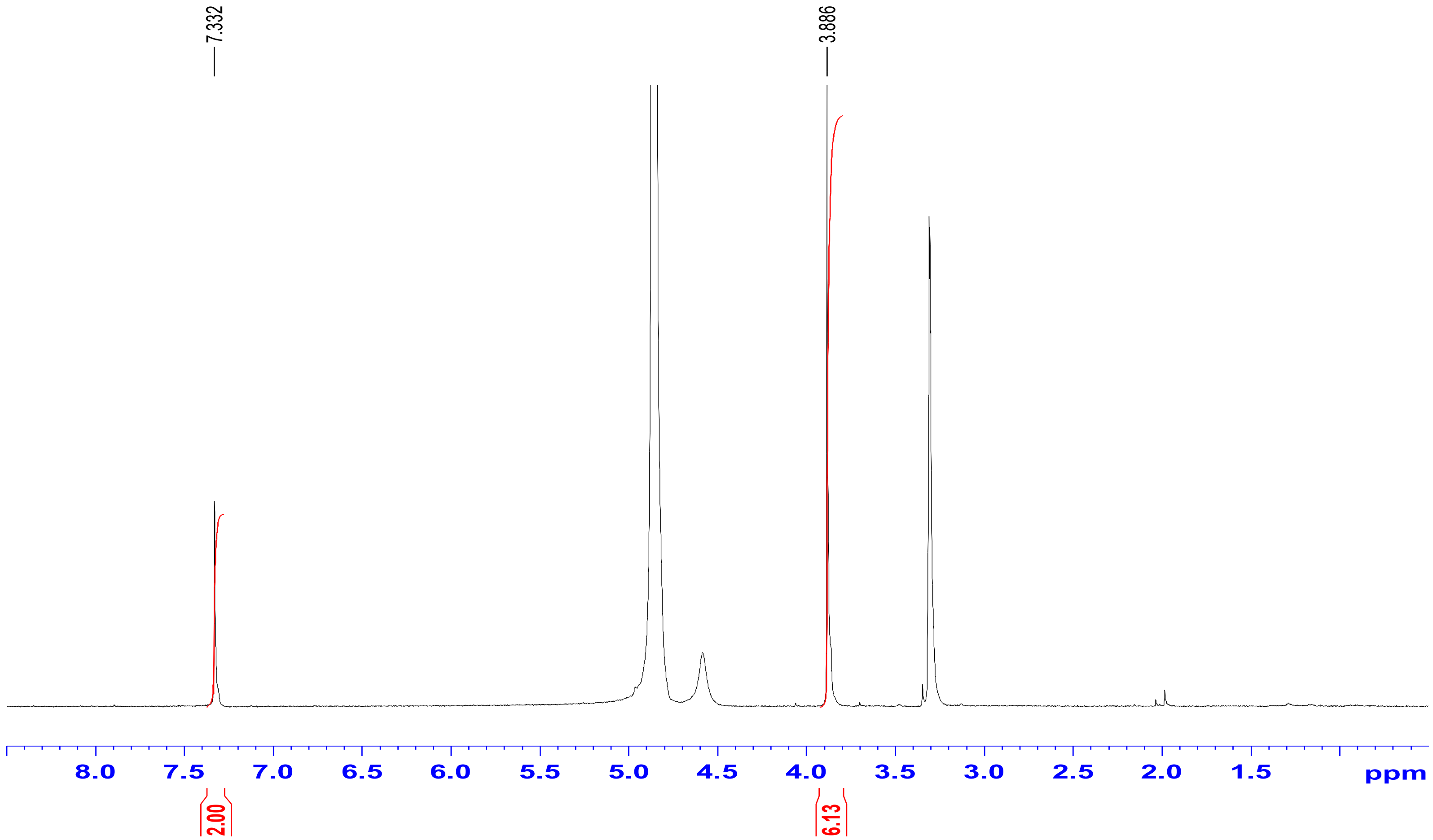

Figure S43. ^{1}H NMR spectrum of **10** in CD_3OD (400 MHz).

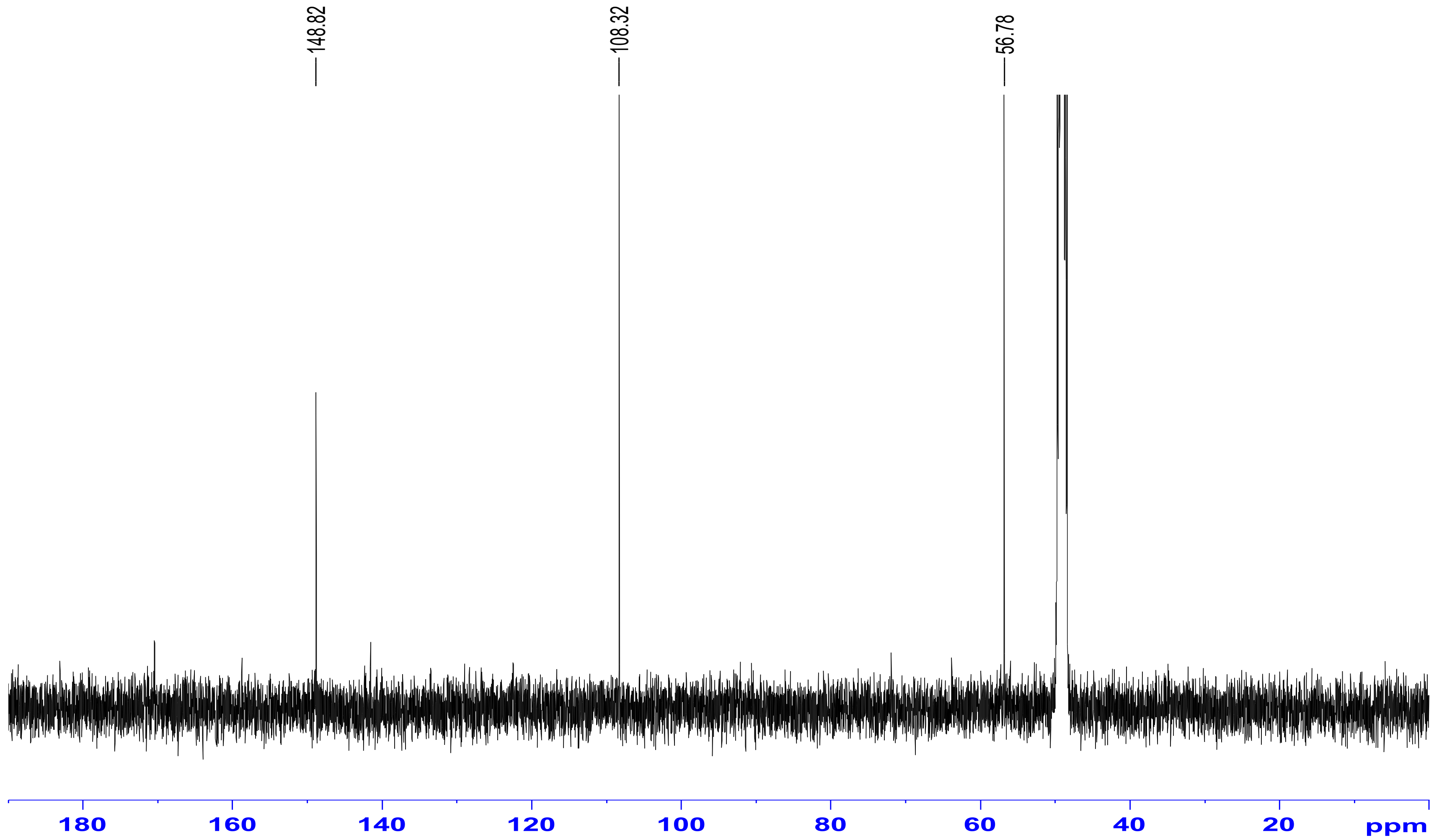

Figure S44. ^{13}C NMR spectrum of **10** in CD_3OD (100 MHz).

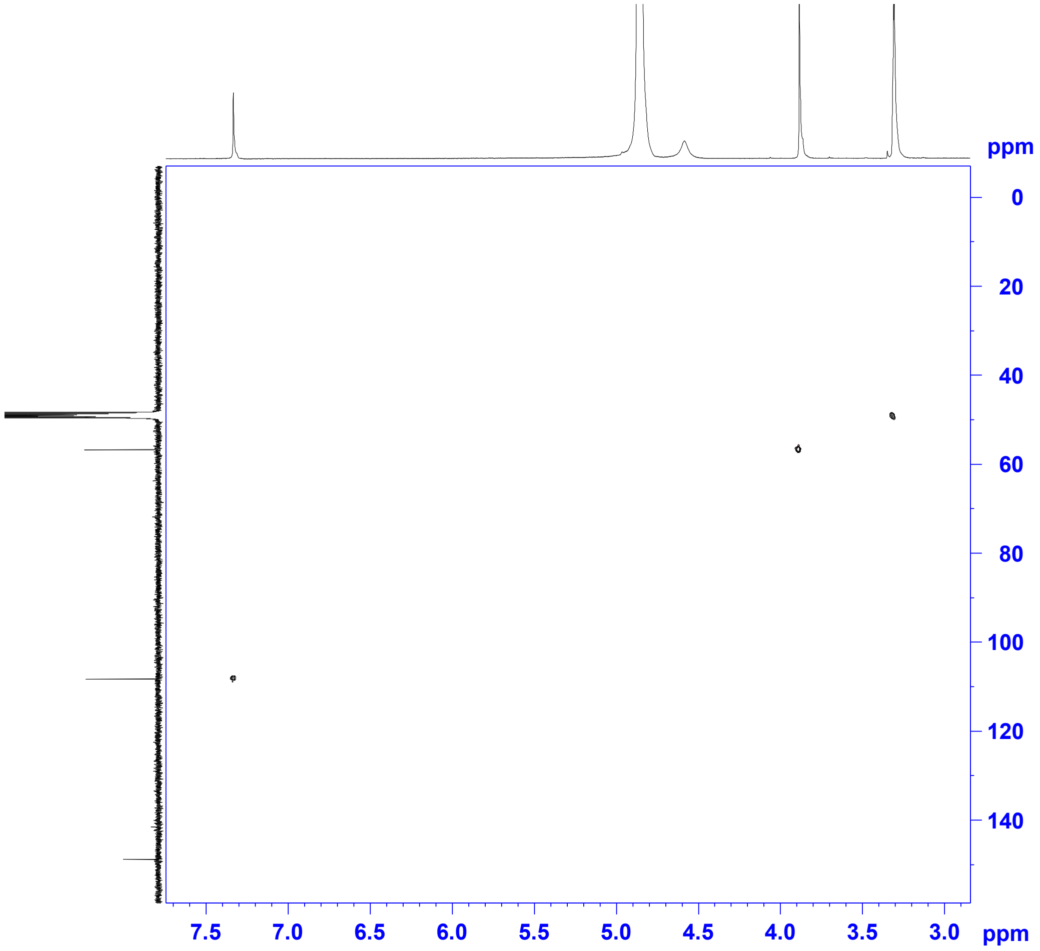

Figure S45. HSQC spectrum of **10** in CD_3OD.

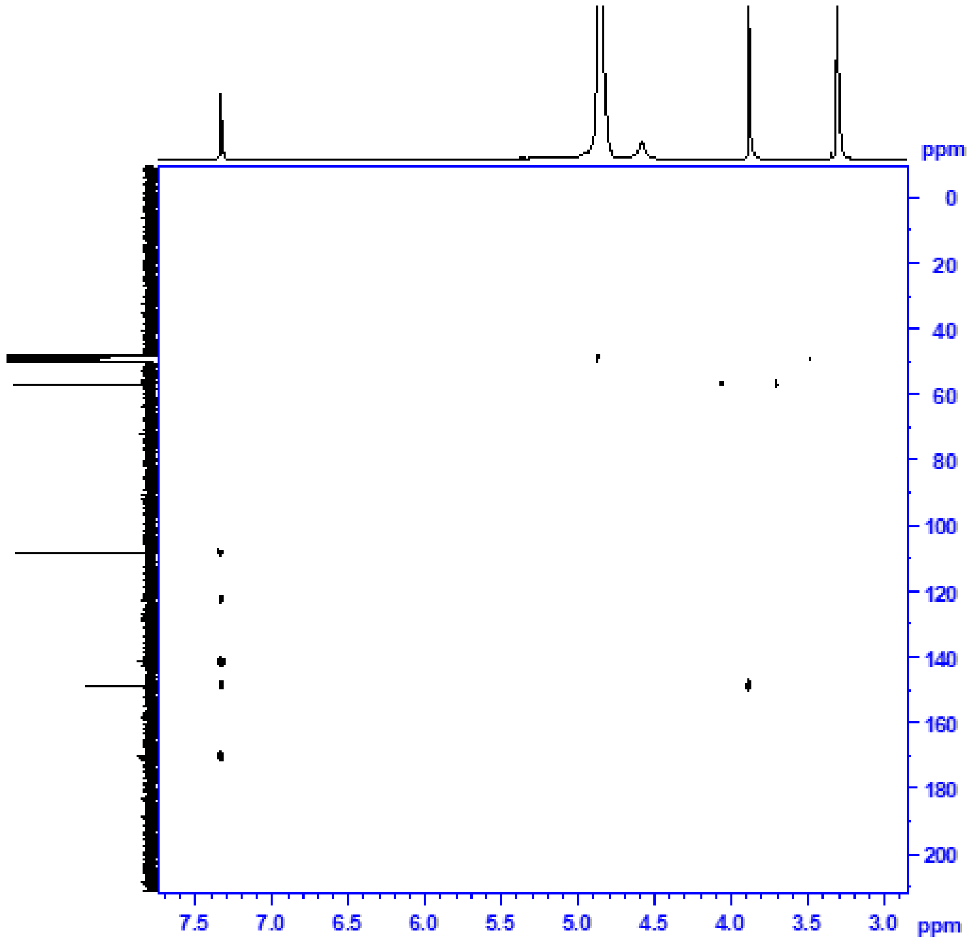

Figure S46. HMBC spectrum of **10** in CD_3OD.
